# Inhibition of RNA polymerase I transcription initiation by CX-5461 activates non-canonical ATM/ATR signaling

**DOI:** 10.18632/oncotarget.10452

**Published:** 2016-07-06

**Authors:** Jaclyn Quin, Keefe T. Chan, Jennifer R. Devlin, Donald P. Cameron, Jeannine Diesch, Carleen Cullinane, Jessica Ahern, Amit Khot, Nadine Hein, Amee J. George, Katherine M Hannan, Gretchen Poortinga, Karen E. Sheppard, Kum Kum Khanna, Ricky W. Johnstone, Denis Drygin, Grant A. McArthur, Richard B. Pearson, Elaine Sanij, Ross D. Hannan

**Affiliations:** ^1^ Research Division, Peter MacCallum Cancer Centre, St. Andrews Place, East Melbourne, Victoria, Australia; ^2^ Department of Biochemistry and Molecular Biology, University of Melbourne, Parkville, Victoria, Australia; ^3^ Sir Peter MacCallum Department of Oncology, University of Melbourne, Parkville, Victoria, Australia; ^4^ The John Curtin School of Medical Research, Australian National University, Acton, ACT, Australia; ^5^ Department of Pathology, University of Melbourne, Parkville, Victoria, Australia; ^6^ School of Biomedical Sciences, University of Queensland, Brisbane, Queensland, Australia; ^7^ Department of Medicine, St Vincent's Hospital, University of Melbourne, Fitzroy, Victoria, Australia; ^8^ QIMR Berghofer Medical Research Institute, Brisbane City, Qld, Australia; ^9^ Pimera Inc, San Diego, CA, USA; ^10^ Department of Biochemistry and Molecular Biology, Monash University, Clayton, Victoria, Australia; ^11^ Department of Molecular Biosciences, The Wenner-Gren Institute, Stockholm University, Stockholm, Sweden; ^12^ Institute for Molecular Medicine Finland, Biomedicum 2, Helsinki, Finland; ^13^ Josep Carreras Institute for Leukaemia Research (IJC), Campus ICO-HGTP, Badalona, Barcelona, Spain

**Keywords:** RNA polymerase I, rDNA, CX-5461, nucleolar stress response, DNA damage signaling

## Abstract

RNA polymerase I (Pol I)-mediated transcription of the ribosomal RNA genes (rDNA) is confined to the nucleolus and is a rate-limiting step for cell growth and proliferation. Inhibition of Pol I by CX-5461 can selectively induce p53-mediated apoptosis of tumour cells *in vivo*. Currently, CX-5461 is in clinical trial for patients with advanced haematological malignancies (Peter Mac, Melbourne).

Here we demonstrate that CX-5461 also induces p53-independent cell cycle checkpoints mediated by ATM/ATR signaling in the absence of DNA damage. Further, our data demonstrate that the combination of drugs targeting ATM/ATR signaling and CX-5461 leads to enhanced therapeutic benefit in treating p53-null tumours *in vivo*, which are normally refractory to each drug alone. Mechanistically, we show that CX-5461 induces an unusual chromatin structure in which transcriptionally competent relaxed rDNA repeats are devoid of transcribing Pol I leading to activation of ATM signaling within the nucleoli. Thus, we propose that acute inhibition of Pol transcription initiation by CX-5461 induces a novel nucleolar stress response that can be targeted to improve therapeutic efficacy.

## INTRODUCTION

Transcription of rDNA by RNA polymerase I (Pol I) underpins the structure of the nucleoli, which form around active clusters of rDNA termed nucleolar organizer regions (NORs). The nucleolus coordinates the monitoring and maintenance of ribosome biogenesis, which is tightly linked to nucleolar structure and integrity [1–4]. However, the primary function of the nucleoli is not limited to the production of ribosomal subunits. Bioinformatic analysis of the nucleolar proteome revealed that fewer than half of the 4500 nucleolar proteins are involved in ribosome biogenesis, while the rest include factors associated with mRNA metabolism, chromatin structure, cell cycle control, DNA replication and repair [5, 6]. The nucleolus indirectly, through sequestration and release of these proteins, has the potential to modulate a diverse range of cellular functions including regulation of tumor suppressor and proto-oncogene activities, cell-cycle control and the DNA damage response (DDR). Furthermore, the nucleolar proteome is dynamically altered in response to nutrient/growth factor signaling and stress responses (reviewed in [7–10]).

The nucleolus is the ‘gatekeeper’ of the nucleolar surveillance pathway, also known as the nucleolar stress pathway [1, 2, 4, 8, 11–13]. The canonical nucleolar stress pathway is induced following acute insults that inhibit ribosome biogenesis and is associated with accumulation of the tumour suppressor protein p53, which mediates cell cycle arrest, senescence or apoptosis depending on the cellular context [14–21]. Therapeutic targeting of p53 through perturbation of ribosome biogenesis is thought to be a mechanism utilized by many cancer therapeutic drugs [22, 23]. Furthermore, we have shown that selective inhibition of ribosomal RNA (rRNA) gene transcription is a new potential paradigm for cancer therapy and a specific small molecule inhibitor of Pol I transcription initiation (CX-5461) has progressed to clinical trial [10, 21, 24–29].

Indeed, we previously demonstrated that CX-5461 selectively induces nucleolar stress resulting in p53-mediated apoptosis of MYC-driven B-lymphoma cells *in vivo* with minimal effects on wild type cells of the same lineage [21, 25]. The canonical nucleolar stress pathway, initiated following disruption of ribosome biogenesis, is characterized by the release of ribosomal proteins (RPs), predominantly RPL5 and RPL11, from the nucleolus, which bind MDM2 and thereby relieves its inhibitory activity towards p53 [1, 30, 31].

While the therapeutic efficacy of Pol I inhibition in B-cell lymphomas has been linked to p53 activation [21, 25], we have demonstrated in earlier studies that the efficacy of CX-5461 in solid tumours is independent of p53 and is associated with cell cycle arrest, senescence and autophagy [32]. Recently, CX-5461 was reported to induce p53-independent G2 checkpoint and apoptosis mediated by the Ataxia telangiectasia mutated (ATM) and Ataxia telangiectasia and Rad3 related (ATR) kinase pathway in acute lymphoblastic leukemia [33, 34]. In this paper, we extend these findings by examining the mechanisms underlying the p53-independent cellular response to Pol I transcription inhibition by CX-5461 to further understand its potential to target solid tumours and to identify targets for rational combination therapies to improve the therapeutic efficacy of targeting Pol I transcription.

We used primary immortalized human fibroblasts (BJ-T) and BJ-T cells stably expressing a short hairpin RNA (shRNA) targeting p53 (BJ-T p53sh), to examine in detail, the biological consequences of inhibiting Pol I transcription initiation in primary cells lacking functional p53. Both BJ-T and BJ-T p53sh cell lines undergo a G1 and G2 cell cycle arrest and senescence in response to CX-5461 treatment. We demonstrate that CX-5461 activates ATM and ATR kinase signaling in the absence of global DNA damage. We further demonstrate that inhibition of ATM/ATR-mediated cell cycle arrest leads BJ-T p53sh and an isogenic RAS and SV40-transformed cell line (BJ-LSTR) to undergo mitotic catastrophe and subsequent CX-5461-mediated cell death *in vitro* and improves the therapeutic efficacy of CX-5461 in targeting aggressive *Tp53*-null (*Tp53*^−/−^) MYC-driven lymphomas *in vivo*.

Active rRNA genes are among the most highly transcribed genes in the genome and are normally characterized by high Pol I loading density [35]. Mechanistically, our data demonstrate that CX-5461 prevents Pol I loading on the rDNA leading to abnormal open and accessible rDNA repeats devoid of Pol I, which is associated with activation of ATM signaling within the nucleoli in the absence of DNA damage. We therefore propose that perturbations of rDNA chromatin trigger a previously unrecognised nucleolar stress response that activates a non-canonical DDR. Importantly, the combination of CX-5461 and inhibition of DDR leads to enhanced therapeutic efficacy in treating an aggressive *Tp53*-null Eμ-*Myc* lymphoma *in vivo.*

## RESULTS

### Inhibition of Pol I transcription by CX-5461 induces p53-independent G1 and G2 checkpoints

**Figure 1:**
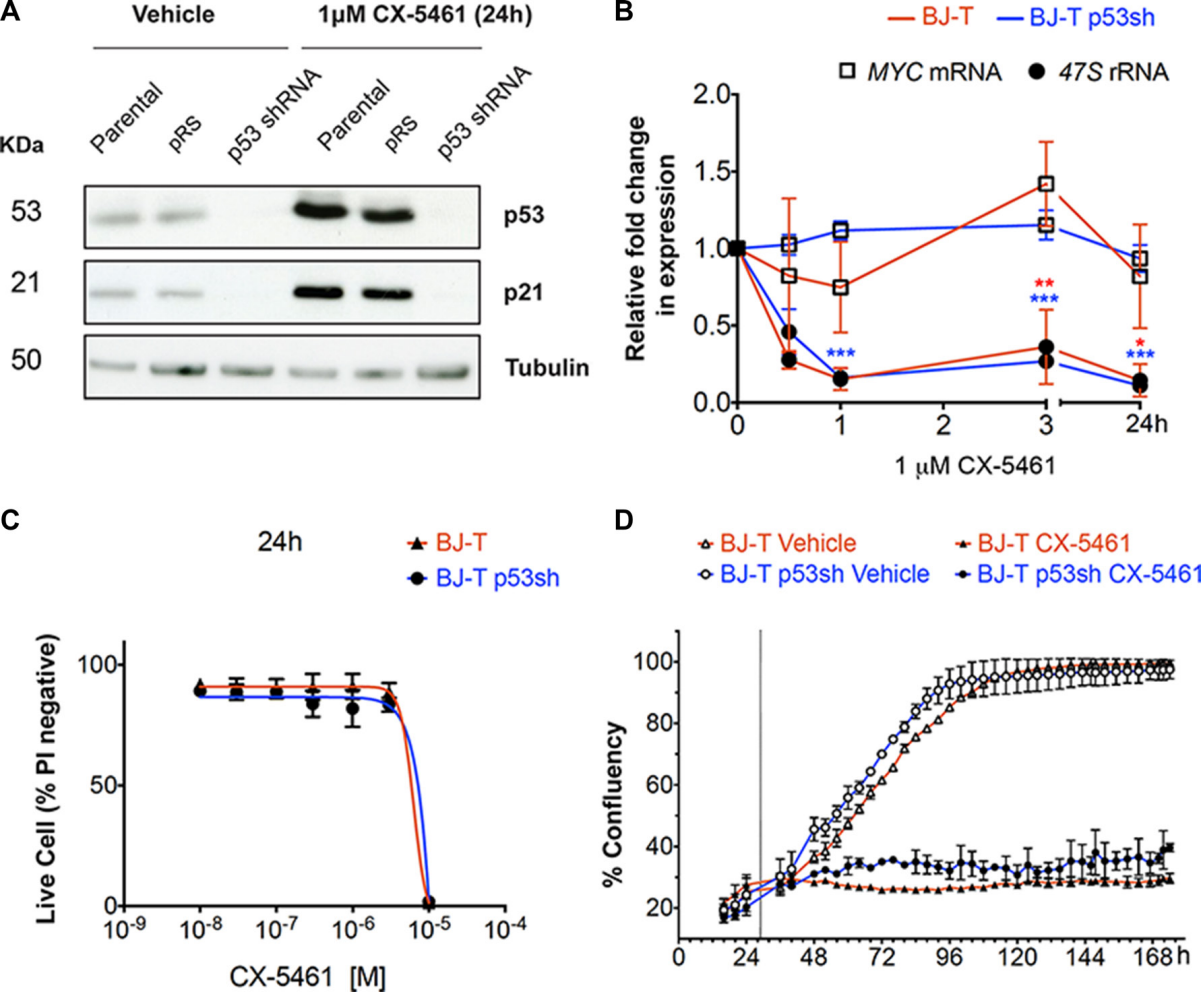
BJ-T fibroblasts undergo p53-independent proliferation defect following inhibition of Pol I transcription initiation by CX-5461 (**A**) Western blot analysis of p53, p21 and tubulin protein levels in parental BJ-T cells and BJ-T cell lines transduced with empty vector (pRS) or p53 shRNA-pRS treated with 1 μM CX-5461 for 24 h (representative of *n* = 3). (**B**) BJ-T (red line) and BJ-T p53sh cells (blue line) were treated with either vehicle or 1 μM CX-5461 for the indicated times. RNA was extracted and the levels of 47S rRNA precursor (dark circle) and *MYC* mRNA (empty square) were determined using reverse transcription qPCR. Expression levels were normalized to Vimentin mRNA and expressed as fold change relative to vehicle *t* = 0 (*n* = 3), error bars represent mean ± s.e.m, *p-value < 0.05, **p-value < 0.01, ***p-value < 0.001, compared to vehicle *t* = 0 samples. (**C**) Propidium iodide (PI) exclusion assay to determine the percentage (%) of live cells of the BJ-T (*n* = 2) and BJ-T p53sh (*n* = 2) cell lines treated with CX-5461 as indicated. Error bars represent mean ± s.d. (**D**) Proliferation time course of BJ-T and BJ-T p53sh cell lines determined by percentage confluency using IncuCyte ZOOM of the BJ-T and BJ-T p53sh cell lines. Dashed line indicates the addition of vehicle or 1 μM CX-5461. Error bars represent mean ± s.d. of 2 technical replicates (representative of *n* = 6).

To understand the mechanisms underlying the biological response to inhibition of Pol I transcription initiation including p53-independent responses, we examined the cellular effect of CX-5461 on primary TERT-immortalized human foreskin fibroblasts (BJ-T) and BJ-T cells stably expressing p53 shRNA (BJ-T p53sh) (Figure 1A). Treatment of BJ-T and BJ-T p53sh cells for 1 hour (h) with 1 μM CX-5461 reduced Pol I transcription initiation rates by approximately 90% (Inhibitory Concentration 90% (IC_90_)) (Figure 1B). Consistent with our previous studies [21, 32], at this drug concentration we observed no effect on Pol II-mediated transcription of *MYC,* whose mRNA half-life (~35 minutes) is similar to pre-rRNA (Figure 1B), thus demonstrating the selectivity of CX-5461 for Pol I- versus Pol II-mediated transcription. Our previous reports using lymphoma cells demonstrated that inhibition of Pol I transcription initiation by CX-5461 led to induction of p53 protein levels and p53-mediated apoptosis [21, 25]. We therefore examined the effect of CX-5461 on p53 levels in BJ-T cells as well as the knockdown levels of p53 in p53sh cells after 24 h of CX-5461 treatment (Figure 1A). CX-5461 led to p53 protein stabilization and induced expression of its transcriptional target p21 while no induction in p53 and p21 protein levels were detected in BJ-T p53sh cells confirming the efficacy of p53 knockdown (Figure 1A). Treatment of BJ-T cells with 1 μM CX-5461 did not induce cell death (Figure 1C) but instead caused a pronounced decrease in cell proliferation (Figure 1D) consistent with the activation of p53 and increased p21 expression (Figure 1A). BJ-T p53sh cells also exhibited a proliferation defect in response to 1 μM CX-5461, consistent with our observed p53-independent CX-5461-mediated growth inhibitory responses in solid tumor cell lines [32]. After chronic treatment with CX-5461, both BJ-T and BJ-T p53sh cells displayed markers associated with senescence including flattened morphology and increased β-galactosidase staining (Figure S1A and S1B). Thus, inhibition of Pol I transcription initiation by CX-5461 in primary cells leads to senescence, which occurs independently of p53 status.

**Figure 2:**
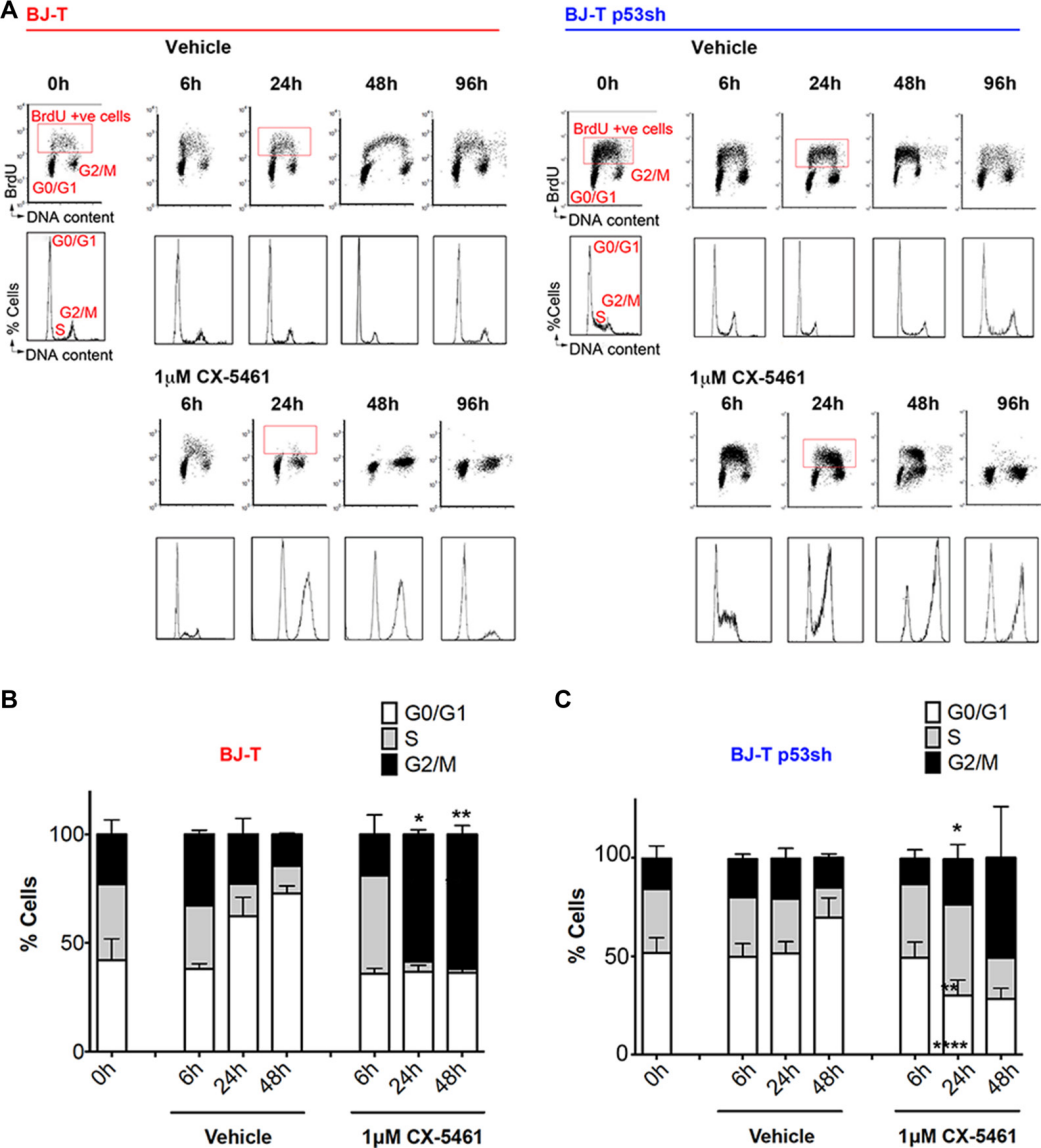
BJ-T and BJ-T p53sh cells exhibit G1 arrest, S-phase delay and G2 cell cycle arrest in response to CX-5461 (**A**) Cell Cycle analysis of BJ-T (left panel) and BJ-T p53sh (right panel) treated with vehicle or 1 μM CX-5461 for the indicated times and labeled with BrdU for 30 min prior to harvest. (Top Panel) Analytical FACS analysis showing BrdU incorporation as a function of DNA content. The boxes represent S-phase BrdU-labeled populations. G0/G1 and G2M cell populations are indicated. (Bottom panels) Histogram plots for total cells stained with PI from the corresponding BrdU-labeled cells shown in top panels. Cell cycle analysis using PI for DNA content of BJ-T (**B**) BJ-T p53sh cells (**C**) treated with CX-5461 as indicated and stained with PI. The percentage of cells in G0/G1, S and G2/M phases were determined using Modfit 3.0 software. BJ-T cells displayed a significant increase in G2/M cells following 24 h to 48 h of treatment with CX-5461 relative to corresponding vehicle treated samples (*n* = 3), error bars represent mean ± s.e.m, *p < 0.05, **p-value < 0.01. BJ-T p53sh cells displayed significant increases in S-phase cells (**p-value < 0.01) and G2/M (*p < 0.05) as well as significant decreases in the G1 populations (****p-value < 0.0001) following 24 h treatment with CX-5461 relative to vehicle treated samples (*n* = 6), error bars represent mean ± s.e.m.

To examine in detail the consequences of inhibiting Pol I transcription initiation on the cell cycle we examined cell cycle progression after 6, 24 and 48 and 96 h of CX-5461 treatment and evaluated cell cycle progression using BrdU incorporation (Figure 2A). Both vehicle and CX-5461-treated BJ-T cells exhibited higher proportion of cells in G1 over time due to confluence induced growth arrest (Figure 2B). However, CX-5461-treated BJ-T cells exhibited a delay in S–phase progression within 6 h of treatment and a halt in BrdU incorporation by 24 h with cells accumulating in the G1 and G2 phases of the cell cycle (Figure 2A and 2B). In contrast, BJ-T p53sh cells continued to incorporate BrdU by 24 h and 48 h but exhibited a pronounced S-phase delay and a G2 cell cycle arrest by 96 h (Figure 2A). The increase in G2-arrested cells at 24 h of CX-5461 treatment is concomitant with a significant decrease in the percentage of cells accumulating in G1 (Figure 2C). Furthermore, both BJ-T and BJ-T p53sh cells treated with CX-5461 for 24 h did not show an increase in histone H3 phosphorylation on serine 10 even when BJ-p53sh were treated with CX-5461 in the presence of nocodazole suggesting that they arrest in the G2 checkpoint before entry into mitosis (Figure S1C and S1D). The data suggest that p53 depletion allows the cells, at least partially, to bypass the G1 checkpoint before arresting in G2 in a p53-independent manner. Nevertheless, a distinct pool of BJ-T p53sh cells remained arrested in G1, suggesting additional p53-independent mechanisms underlying the G1 arrest in response to acute inhibition of Pol I transcription initiation by CX-5461. Taken together these data demonstrate that while CX-5461 induces a p53-mediated G1 checkpoint, it also activates p53-independent G1, S-phase and G2 checkpoints.

### CX-5461 activates ATM/ATR signaling pathways independent of p53

To explore the mechanisms underlying the CX-5461-mediated G2 cell cycle arrest, we examined the activity of the G2 checkpoint kinases ATM and ATR. ATM phosphorylates multiple substrates including the histone variant H2AX on serine 139 (γH2AX), CHK2 on threonine 68 and p53 on serine 15. Phosphorylation of H2AX signals that DNA damage has occurred and is required for the assembly of proteins associated with DDR (Reviewed in [36, 37]). Once activated, CHK2 phosphorylates many substrates involved in cell cycle progression including p53 and the CDC25 family of phosphatases. CHK2 inactivates the CDC25 phosphatases, leading to the maintenance of inhibitory phosphorylation (tyrosine 15) of cyclin dependent kinases (CDKs) such as CDK2 and CDK1 and the initiation of S and G2 cell cycle checkpoints. The major substrate of ATR is CHK1, which upon activation by phosphorylation at serine residues 317 and 345 also phosphorylates the CDC25 phosphatases (Reviewed in [37]).

**Figure 3:**
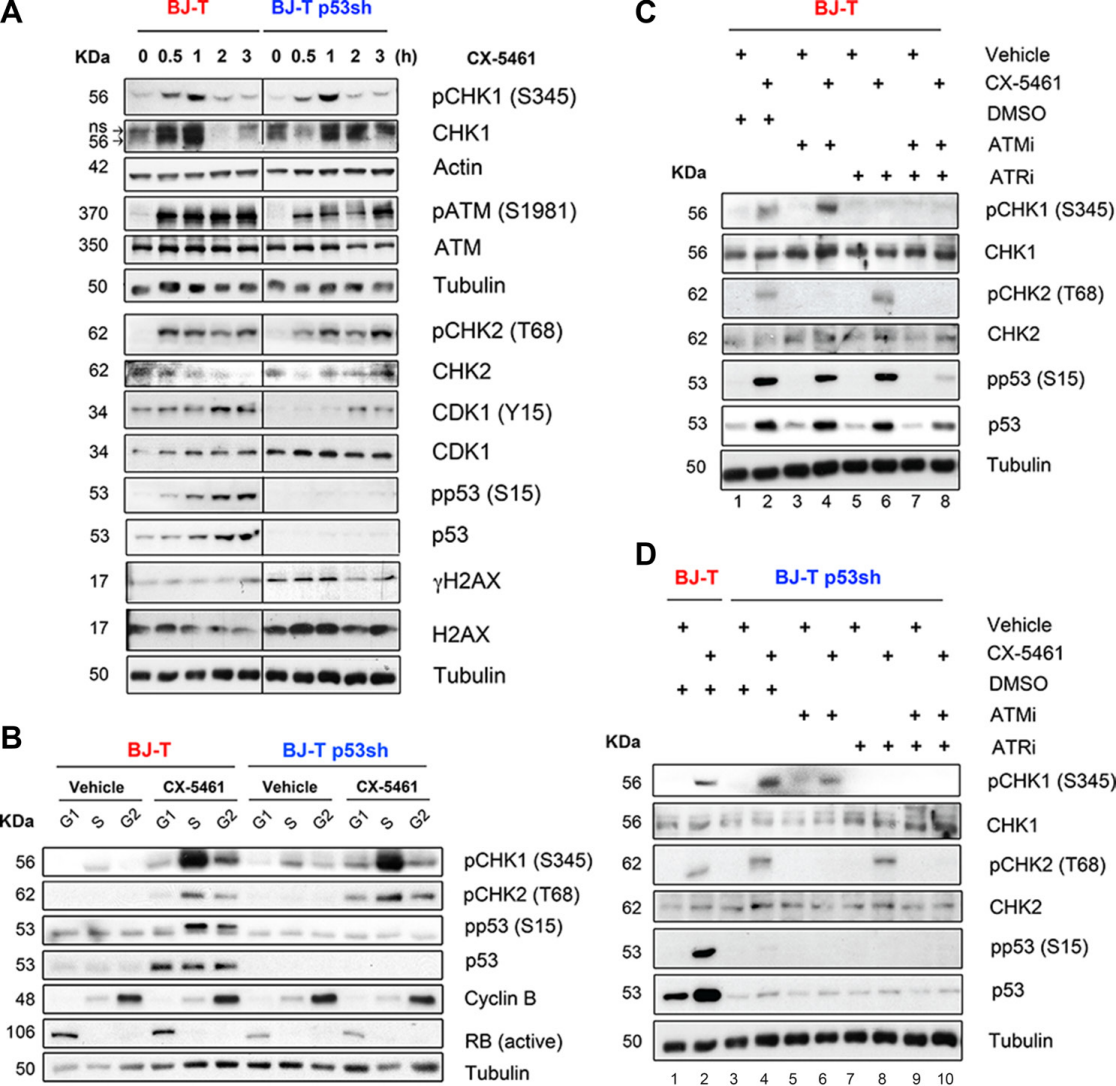
Inhibition of Pol I transcription initiation by CX-5461 activates the ATM/ATR signaling pathway (**A**) Western blot analysis of BJ-T and BJ-T p53sh cell lines treated with either vehicle (0 h) or 1 μM CX-5461 for the indicated time course. Total protein lysates were extracted and western blot analysis was carried out with the indicated antibodies against phosphorylated and total ATM, CHK1, CHK2, CDK1 and H2AX proteins and antibodies that detect actin and Tubulin protein levels. (**B**) BJ-T and BJ-p53sh cells were treated with either vehicle or 1 μM CX-5461 for 2 h then collected and incubated with Vybrant DyeCycle Violet stain (Life Technologies). Cells were sorted using BD FACS Aria, into G1 and S phase and G2/M populations based on DNA content and total protein lysates were extracted. Western blot analysis was performed to examine phosphorylation levels of p53, CHK1 and CHK2 proteins as well as total p53 and Tubulin. Active RB and Cyclin B levels were used as quality control markers for G1 and G2 populations. The reduction in active RB levels in BJ-T p53shRNA cells is likely due to the fact that RB is a transcriptional target of p53 [83]. (**C**) BJ-T and (**D**) BJ-T p53sh cells were pretreated for 30 min with either DMSO, 5 μM KU-5593 (ATMi), 5 μM VE-821 (ATRi) or 5 μM ATMi/ 5 μM ATRi before vehicle or 1 μM CX-5461 were added to the cultures for further 2 h. Western blot analysis for phosphorylated and total CHK1, CHK2 and p53 were performed. All experiments in A-D are representatives of *n* = 3.

To investigate acute responses to CX-5461, BJ-T and BJ-T p53sh cells were treated with 1 μM CX-5461 and activation of ATM/ATR pathway signaling was examined at 30 minutes (min), 1, 2 and 3 hours (Figure 3A). Increases in the abundance of phosphorylated CHK1 (S345), ATM (S1981) and CHK2 (T68) and total CHK1 were detected by 30 min, which was associated with an increase in the inhibitory CDK1 (Y15) phosphorylation that is known to be involved in initiating the G2 checkpoint (Figure 3A). Intriguingly, total CDK1 levels were induced in BJ-T p53sh cells compared to BJ-T cells in agreement with previous reports demonstrating that p53 negatively regulates CDK1 expression [38]. Nevertheless BJ-T p53sh cells underwent p53-independent G2 cell cycle arrest (Figure 2A and 2C). CX-5461 also induced phosphorylation of p53 on serine 15, which is a direct substrate of ATM and ATR [39–42]. However, CX-5461 mediated activation of ATM and ATR signaling in BJ-T and BJ-T p53sh cells occurs independently of increases in γH2AX levels, a marker of DNA double strand breaks. Although basal H2AX and γH2AX levels are induced in BJ-T p53sh cells in agreement with p53′s role in regulating H2AX levels [43], no further induction in γH2AX levels was detected following acute CX-5461 treatment, indicating that CX-5461 mediated activation of ATM/ATR signaling occurs in the absence of global DNA damage and independently of p53.

To determine the contribution of ATM and ATR signaling to CX-5461-mediated G1, S and G2 checkpoints, we examined CHK1, CHK2 and p53 phosphorylation in fluorescence-activated-cell sorted (FACS) sorted G1, S and G2 populations (Figure 3B). BJ-T and BJ-T p53sh cells were treated with vehicle or 1 μM CX-5461 for 2h at which time point optimal p53 phosphorylation at S15 as well as robust levels of CHK1 (S345) and CHK2 (T68) phosphorylation were observed (Figure 3A). In BJ-T cells, phosphorylation of the ATR target CHK1 at S345 occurred predominantly in S phase and to lesser extent in G1 and G2, while CHK2 (T68) and p53 (S15) phosphorylation were detected mainly in the S and G2 populations (Figure 3B). In contrast, p53 protein levels increased at all stages of the cell cycle following CX-5461 treatment (Figure 3B). Therefore, the data suggest a role for p53 in mediating the G1 arrest while ATM and ATR mediate the S-phase delay and the G2 arrest in BJ-T cells in response to CX-5461. In BJ-T p53sh cells, CX-5461 induced an increase in phosphorylated CHK2 (T68) at all stages of the cell cycle while CHK1 (S345) phosphorylation was predominant in the S phase population and to a lesser extent in the G1 and G2 pools. Thus, in BJ-T p53sh cells, ATM and ATR activity is associated with CX-5461-mediated S-phase delay while ATM activity correlated with CX-5461-induced p53-independent G1 and G2 arrest (Figure 2B).

To further define the relative roles of ATM and ATR in CX-5461-mediated cell cycle defects, we pre-treated BJ-T (Figure 3C) and BJ-T p53sh cells (Figure 3D) with ATM (KU-55933, ATMi) and ATR (VE-821, ATRi) inhibitors alone or in combination for 30 min followed by 2h of CX-5461. Pre-treatment with ATMi completely abolished CX-5461-mediated CHK2 (T68) phosphorylation but not CHK1 (S345) phosphorylation in both cell types (Figure 3C, lane 2 versus 4; Figure 3D, lane 4 versus 6). Conversely, pre-treatment with the ATRi prevented CX-5461-mediated CHK1 (S345) phosphorylation but did not affect the abundance of phosphorylated CHK2 (T68) (Figure 3C, lane 2 versus 6; Figure 3D, lane 4 versus 8). In BJ-T cells, p53 (S15) phosphorylation was attenuated after combined inhibition of ATM and ATR (Figure 3C, lane 2 versus 8). Thus, in response to inhibition of Pol I transcription initiation by CX-5461 ATM and ATR are independently activated upstream of p53 (Figure 3C).

**Figure 4:**
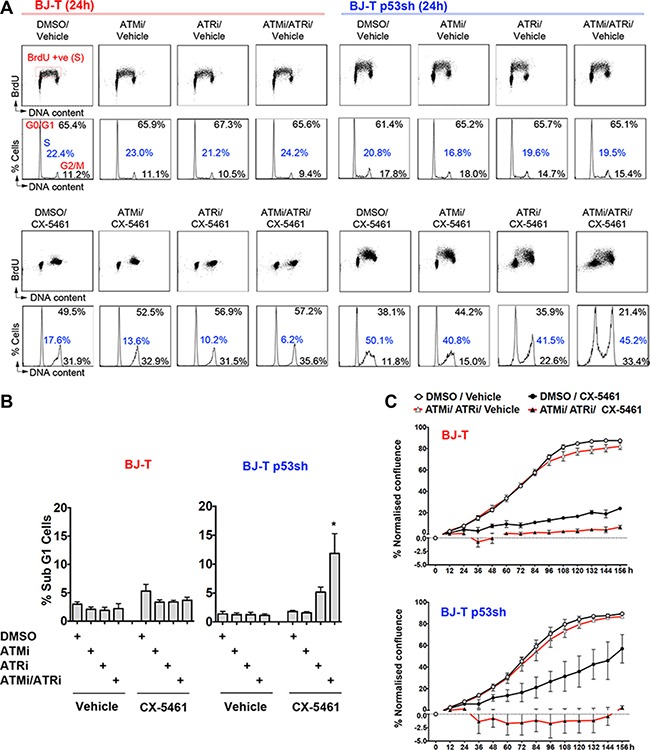
Combination treatment of ATM and ATR inhibitors with CX-5461 induces cell death in the absence of p53 (**A**) Cell Cycle analysis of BJ-T cells (left panel) and BJ-T p53sh cells (right panel) following 24 h of treatment with either vehicle or 1 μM CX-5461 in combination with either DMSO, 5 μM ATMi, 5 μM ATRi or 5 μM ATMi/ 5 μM ATRi. BrdU incorporation analysis (upper panels) and PI staining for DNA content (lower panels) were performed as described in Figure 2A. The percentages of live cells in G0–G1, S, and G2–M phases were determined using Modfit 3.0 software (representatives of *n* = 3). (**B**) Quantitation of cell death from (A), determined by subG1 DNA content analysis using FCS Express software (*n* = 3), error bars represent mean ± s.e.m, *p-value < 0.05 relative to CX-5461 treated BJ-T p53sh cells. (**C**) Proliferation time course using IncuCyte ZOOM of BJ-T and BJ-T p53sh cells treated with vehicle or 100 nM CX-5461 in the presence of DMSO or (5 μM ATMi/5 μM ATRi). Confluency values was normalised to percentage confluency at time 0h (*n* = 3), error bars represent mean ± s.e.m.

We then investigated whether ATMi, ATRi or the combination of ATMi/ATRi could abrogate CX-5461-induced cell cycle checkpoints. Co-treatment of CX-5461 with ATMi or ATRi alone had no effect on the CX-5461-mediated G1 or the G2 arrest but partially relieved the S-phase delay of BJ-T and BJ-T p53sh cells (Figure 4A). However, dual ATM and ATR inhibition led BJ-T p53sh cells to bypass the G1 arrest and resulted in cell death, as demonstrated by the increase in the Sub-G1 DNA content fraction (Figure 4B) and the decrease in cell confluence in proliferation assays (Figure 4C). CX-5461-mediated cell death was associated with an increase in the proportion of cells displaying abnormal nuclei and genomic instability (Figure S2A, S2B, S2D and S2E). Thus, dual ATM and ATR inhibition in combination with CX-5461 in BJ-T p53sh cells promoted mitotic catastrophe (Figure S2A, S2B, S2D and S2E). While, BJ-T cells remained arrested following combined CX-5461 and ATM/ATR inhibition most likely due to activation of p53 through the canonical nucleolar stress pathway, BJ-T p53sh cells underwent abnormal mitosis and cell death in response to the abrogation of the ATM/ATR-mediated cell cycle checkpoints (Figure 4B and 4C; Figure S2).

### Inhibition of ATM/ATR signaling sensitizes cancer cells to CX-5461 mediated cell death

To compare the biological response to inhibition of Pol I transcription initiation of cancer cells to those of primary cell, we utilized the tumorigenic LSTR cell line, an isogenic BJ-derived cell line expressing SV40 early region (large-T and small-t), *TERT*, and an oncogenic allele of the *HRAS* gene (HRASG12V), which can grow tumours in nude mice [44]. Intriguingly, similar to BJ-T and BJ-T p53sh cells, CX-5461 treatment induced a proliferation defect in the BJ-LSTR cells despite SV40-mediated inactivation of the p53 and retinoblastoma protein (RB) families of tumour suppressors (Figure S3A). Although BJ-LSTR cells exhibited increased G2 population following CX-5461 treatment, they also showed increased ploidy with accumulating N4 and N8 DNA content (Figure S3B and S3C). However, these cells did not proliferate following treatment with CX-5461 (Figure S3A) possibly due to failed cytokinesis, which is consistent with the increase in DNA content per cell (Figure S3B and S3C). Thus in the absence of ongoing rRNA transcription both immortalized and transformed fibroblast are unable to execute normal cell proliferation, suggesting that in the absence of intact G1 and G2 checkpoints inhibition of Pol I transcription initiation induces additional p53 and RB independent checkpoint(s) preventing cell division. Importantly, similar to BJ-T p53sh cells, the BJ-LSTR cells underwent mitotic catastrophe (Figure S2C and S2F) and cell death following combined inhibition of Pol I transcription initiation and ATM/ATR as indicated by the decrease in cell confluency in proliferation assays (Figure S3D). Taken together, the data strongly suggest the inhibition of ATM/ATR pathways sensitizes p53-null cells to CX-5461 induced cell death via mitotic catastrophe following the failure of G1, S and G2 checkpoints.

**Figure 5:**
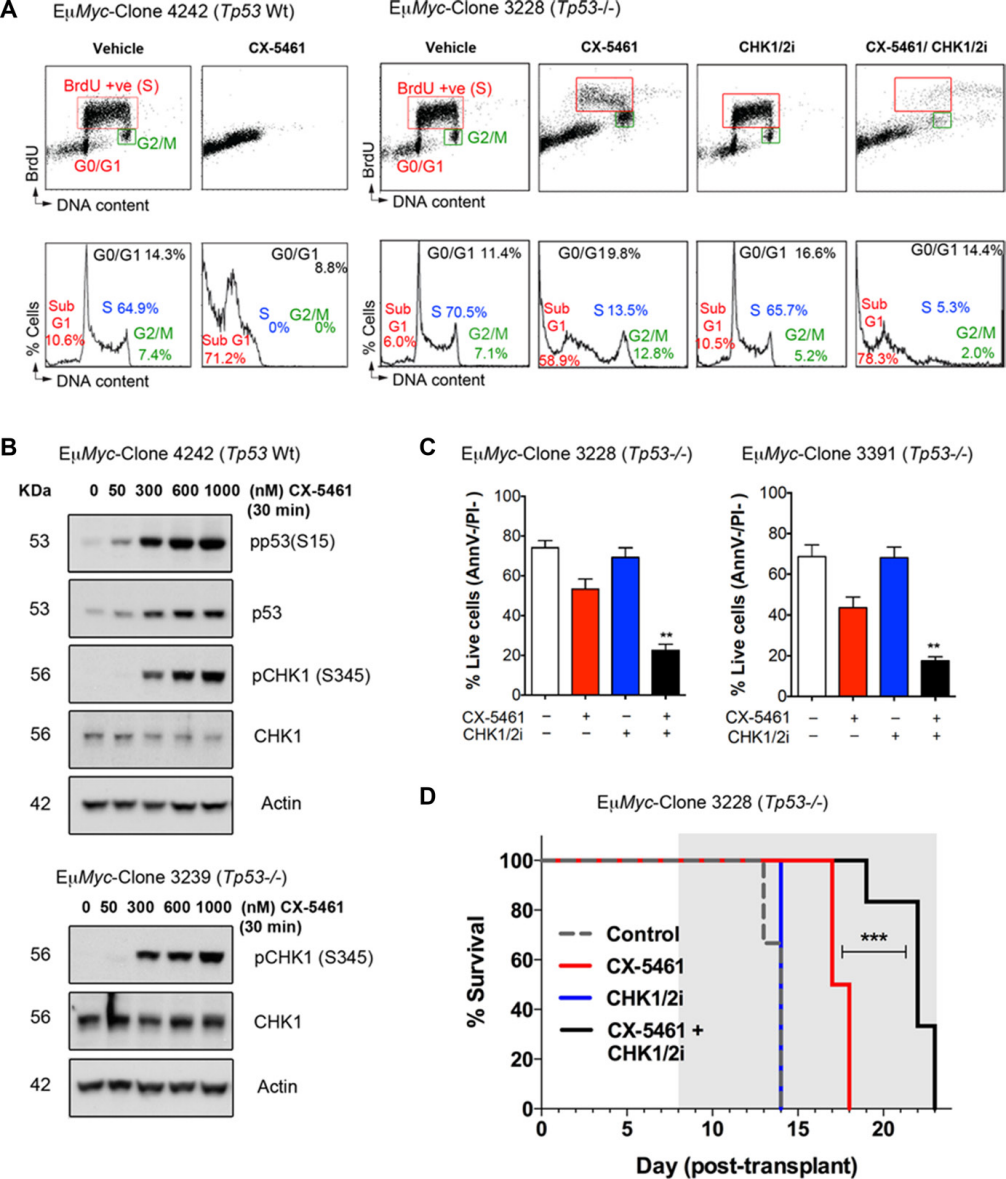
CX-5461 combination with a dual CHK1/CHK2 inhibitor induces cancer cell death of *Tp53*-null (*Tp53*^−/−^ *)* Eμ-*Myc* lymphoma cells *in vitro* and *in vivo* (**A**) Cell cycle analysis using BrdU incorporation (upper panels) and PI staining for DNA content (lower panels) was performed of p53 wild-type (*Tp53* Wt) and (*Tp53*^−/−^) Eμ-*Myc* lymphoma cells following 24-hour treatment with vehicle, 100 nM CX-5461, 30 nM AZD7762 (CHK1/2i) or combined 100 nM CX-5461/30 nM CHK1/2i. Analysis was performed as in Figure 2A (representatives of *n* = 3). (**B**) Western blotting analysis of phosphorylated and total protein levels of p53 and CHK1 in *Tp53* Wt and *Tp53*^−/−^ Eμ-*Myc* lymphoma cells following 30 min treatment with CX-5461 as indicated. Actin levels were used as a loading control. (**C**) Percentages of live *Tp53*^−/−^ Eμ-*Myc* lymphoma cells (Annexin V −ve/ PI −ve) cells treated as in (A) (*n* = 3), error bars represent mean ± s.e.m., **p-value < 0.01 compared to corresponding CX-5461 treated samples. (**D**) CX-5461 combination with CHK1/2i leads to improved therapeutic benefit in treating transplanted *Tp53*^−/−^ Eμ-*Myc* lymphoma cells *in vivo*. Kaplan-Meier curves showing increased survival of mice transplanted with *Tp53*^−/−^ Eμ-*Myc* lymphoma (clone 3228) treated daily with 20 mg/kg AZD7762 (CHK1/2i) every weekday, CX-5461 at 30 mg/kg every third day or combined CX-5461+CHK1/2i (***p < 0.0001; *n* = 6 in every treatment group). Treatment period is indicated by the grey area.

We therefore examined the therapeutic efficacy of combining inhibition of ATM/ATR signaling with CX-5461 in MYC driven B-cell lymphomas (Eμ-*Myc*). Our previous work showed that the therapeutic efficacy of CX-5461 in the Eμ-*Myc* lymphoma model was associated with the presence of a functional p53 pathway [21, 25] (Figure 5A). CX-5461 induced p53 protein levels [21] as well as phosphorylation of p53 (S15) (Figure 5B) in a dose-dependent manner and increased apoptosis of p53 wild-type (*Tp53* Wt) Eμ-*Myc* lymphoma cells (Figure 5A). However, in addition to promoting cell death, as indicated by an elevated proportion of sub-G1 cells, CX-5461 induced G2-arrest in *Tp53*-null (*Tp53*^−/−^*)* Eμ-*Myc* lymphoma cells (Figure 5A). CX-5461 induced CHK1 (S345) phosphorylation in *Tp53* Wt as well as *Tp53*^−/−^ Eμ*Myc* cells (Figure 5B), suggesting CX-5461 activates ATR signaling in a p53 independent manner as observed in the BJ-T p53sh cells (Figure 3). We could not examine the status of pCHK2 (T68) due to the lack of mouse specific anti-CHK2 antibodies.

In order to examine the contribution of ATM/ATR activation to CX-5461-mediated G2 arrest in Eμ-*Myc* lymphoma cells, we combined CX-5461 with a dual CHK1/CHK2 inhibitor (CHK1/2i; AZD7762) and found that CX-5461/CHK1/2i combination abrogates the G2 arrest and renders *Tp53*^−/−^ Eμ-*Myc* cell lines sensitive to cell death (Figure 5A and 5C). We therefore investigated whether this combination could be effective in treating MYC-driven p53 wild type and null lymphoma *in vivo.* In mice transplanted with *Tp53*^−/−^ Eμ-*Myc* B-lymphoma cells, CX-5461 and CHK1/2i as single agents provided a modest survival benefit (median 3.5 days) and no survival benefit, respectively (Figure 5D). However against this aggressive Eμ−*Myc* B-cell lymphoma, the CX-5461/CHK1/2i combination had a significantly improved therapeutic benefit compared to single agent treatment. Thus, G2 checkpoint inactivation, which leads to cell death due to mitotic catastrophe in p53-null cells *in vitro* (Figure S2), can expand the therapeutic efficacy of CX-5461 in targeting cancers lacking functional p53.

### CX-5461 activates ATM in the absence of DNA damage

**Figure 6:**
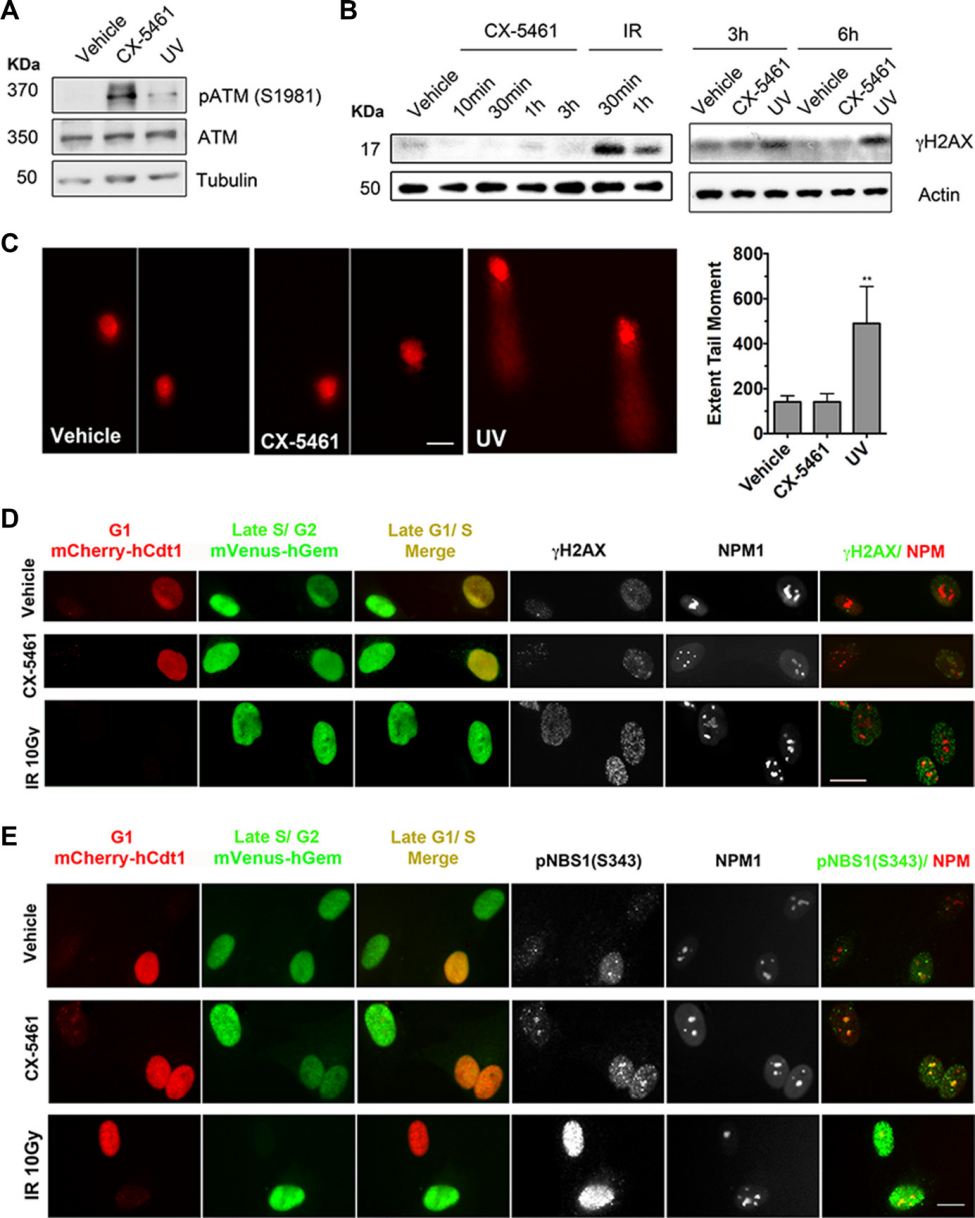
CX-5461 activates ATM signaling within the nucleoli in the absence of DNA damage (**A**) BJ-T cells were treated with vehicle, 1 μM CX-5461 or 100 J/m^**2**^ ultraviolet (UV) irradiation and incubated for 2 h. Total protein lysates were extracted and western blotting analysis of phosphorylated and total ATM and Tubulin levels (Representative o f *n* = 3). (**B**) BJ-T cells were treated with vehicle, 1 μM CX-5461 or 10 Gy ionizing radiation (IR) as indicated. Total protein lysates were extracted and western blotting analysis of γH2AX and Tubulin levels was performed. IR treatment was used as a positive control for induction of γH2AX levels (representative of *n* = 3). (**C**) Alkaline comet assay analysis of DNA damage following 1 μM CX-5461 treatment for 30 min (*n* = 4). UV irradiation (500 J/m^**2**^) followed by 30 min incubation was used as a positive control for induction of DNA damage. Quantitation of extent of tail moment, which is a product of tail length and the percentage of tail DNA was performed using Metamorph software and normalized to vehicle control. Scale bar = 20 μM. Error bars represent mean ± s.e.m., **p < 0.001. (**D**) Co-immunofluorescence analysis of γH2AX and (**E**) pNBS1(S343) with NPM1 in FUCCI-labeled BJ-T p53sh treated with vehicle, 1 μM CX-5461 or 10 Gy IR for 1h. FUCCI cells express a fragment of Cdt1 linked to the fluorescent protein mCherry (monomeric Cherry) during the G1 phase of the cell cycle, as well as a fragment of Geminin linked to the fluorescent protein mVenus (monomeric Venus) during the S/G2/M stages. γH2AX and pNBS1 (S343) overlap with NPM1 in G1/S transition (yellow) or late S/G2/M (green) cells populations were examined in two independent experiments. Scale bar = 10 μM.

ATM and ATR respond to double strand and single strand DNA breaks, DSBs and SSBs respectively. While activation of ATM by autophosphorylation on S1981 was detected following CX-5461 treatment (Figure 6A), phosphorylation of H2AX (S139) was only detected after exposure to ionizing radiation (IR) and UV (Figure 6B). Further, there was no increase in γH2AX foci formation following 30 min of CX-5461 treatment of BJ-T cells (Figure S4A), at which time point robust activation of CHK1 and CHK2 was detected (Figure 3A). To further examine whether CX-5461 might be associated with DNA damage, we used the alkaline comet assay, a sensitive method to detect SSBs, DSBs or base modifications at the single cell level [45]. We found no evidence for DNA damage after 30 min of CX-5461 of BJ-T cells (Figure 6C). In contrast, long comet tails were detected following exposure to UV irradiation. Together, our data suggest that the acute activation of ATM/ATR signaling by CX-5461 occurs in the absence of detectable global DNA damage.

To discount the possibility that our γH2AX IF technique was not sensitive enough to detect DNA damage at the rDNA repeats, we used the doxycycline-inducible U2TR IPpoI-dd U2OS cell line in which DNA damage can be induced at a defined site at the rDNA. IPpoI is an intron-encoded homing endonuclease from the *myxomycete* *Physarum polycephalum* that cuts with high specificity at an endogenous 15 base-pairs (bp) recognition sequence within the 28S transcribed region of the 300 copies of rDNA and up to 13 other sites in the human genome [46, 47]. Induction of IPpoI induced-DSBs at the rDNA (Figure S4B) resulted in nucleolar segregation and enrichment in γH2AX at the nucleolar caps (Figure S4C and S4D), which are characterized by the condensation and separation of the nucleolar compartments and constitute nucleolar proteins such as the upstream binding transcription factor UBF [47, 48]. However, we did not detect γH2AX at the nucleoli following CX-5461 treatment further suggesting that CX-5461 does not induce DSBs at the rDNA (Figure S4C and S4D).

To further investigate the possibility of CX-5461 inducing DNA damage at the rDNA loci in cells at specific stages of the cell cycle, we performed IF analysis of γH2AX combined with nucleophosmin (NPM1) staining, as a marker for the nucleoli, in FUCCI (fluorescent ubiquitinylation cell cycle indicator) [49] labeled BJ-T p53sh cells. We used BJ-T p53sh cells to enrich for the S-phase and G2 populations to examine the presence of γH2AX foci within the nucleoli in late G1-S transition (yellow) or late S-G2/M (green) cell populations following 1h of treatment with CX-5461 (Figure 6D). We found no evidence for global nor nucleoli associated γH2AX foci formation in response to CX-5461 treatment of S and G2 cells as compared to IR-induced γH2AX foci (Figure 6D, Figure S5A). Therefore, our data suggest that acute ATM/ATR activation in response to CX-5461 occurs independently of DNA breaks. Differential activation of ATM substrates has been reported under conditions of non-canonical activation of ATM such as oxidative stress [50, 51]. Further, ATM's recruitment to chromatin, which is mediated by the MRE11/RAD50/NBS1 complex, has been reported to initiate DNA damage signaling without actual DNA damage [52]. We therefore performed IF analysis of phosphorylated Nijmegen Breakage Syndrome 1 (NBS1) at (S343), a component of the MRE complex and a substrate for ATM kinase activity [53]. Unlike γH2AX, pNBS1 (S343) was detected within the nucleoli following 1h of CX-5461 treatment in S-phase cells (Figure 6E, Figure S5B). Thus, the data strongly suggest that inhibition of Pol I transcription initiation by CX-5461 leads to DNA damage-independent activation of ATM signaling in the nucleoli.

### Inhibition of rRNA synthesis *per se* does not activate the ATM/ATR pathway

**Figure 7:**
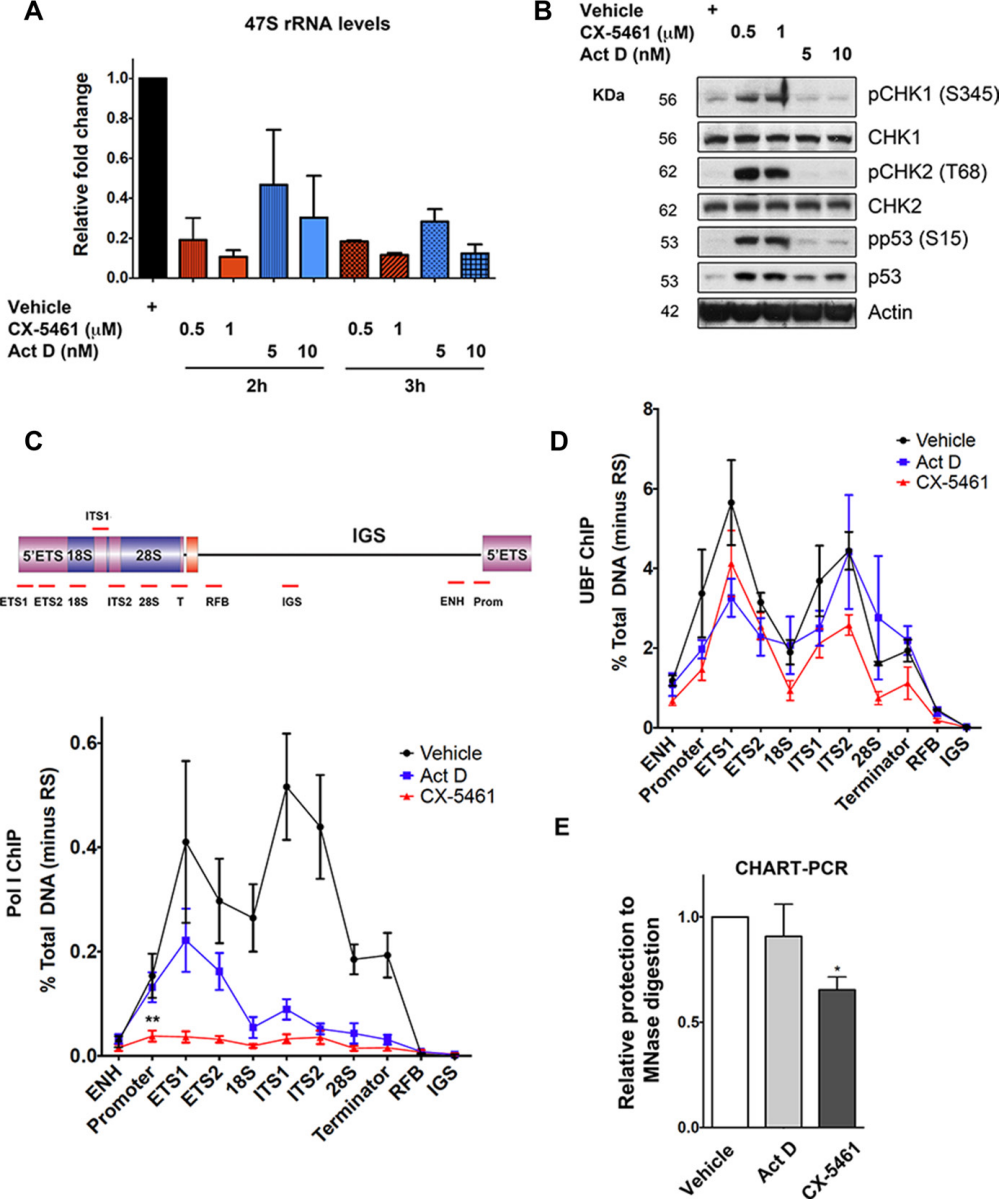
Inhibition of Pol I transcription initiation by CX-5461 results in rDNA repeats that are devoid of Pol I, but maintain an exposed chromatin state that associates with ATM/ATR pathway activation (**A**) BJ-T p53sh cells were treated with vehicle, CX-5461 or Act D as indicated. RNA was extracted and 47S rRNA precursor levels were determined. Expression levels were normalized to Vimentin mRNA and expressed as fold change relative to vehicle *t* = 0 (*n* = 3), error bars represent mean ± s.e.m, *p-value < 0.05, **p-value < 0.01, compared to vehicle *t* = 0 samples. (**B**) Total protein lystaes of BJ-T and BJ-T p53sh cells treated as indicated were extracted and western blot analysis was performed to examine phosphorylation levels of p53, CHK1 and CHK2 proteins as well as total p53 and Tubulin (representative of *n* = 3). (**C**) Schematic of a human rDNA repeat and the positions of qPCR amplicons. (ENH, enhancer; ETS' external transcribed spacer; ITS, internal transcribed spacer; T, terminator; RFB, replication fork barrier; IGS, intergenic spacer). qChIP analysis of Pol I (POLR1A subunit) binding to rDNA in BJ-T p53sh cells treated as in (B) (*n* = 3), error bars represent mean ± s.e.m, **p-value < 0.01, compared to vehicle sample. (**D**) qChIP analysis of UBF binding to rDNA in BJ-T p53sh cells treated with vehicle, 1 μM cx-5461 (2 h) or 5 nM Act D (3 h) (*n* = 3), error bars represent mean ± s.e.m. (**E**) Nuclei from BJ-T p53sh cells treated as in (D) were extracted and incubated with or without MNase. Extracted gDNA was subjected to qPCR using primers targeting the rDNA promoter. MNase accessibility were normalized to undigested gDNA samples and expressed as fold change relative to vehicle (*n* = 3), error bars represent mean ± s.e.m, *p–value < 0.05.

In order to further understand the p53-independent global biological response to CX-5461-mediated nucleolar stress at the transcriptional level, we performed high throughput RNA-sequencing (RNA-seq) analysis following a time course (30 min, 1 h, 3 h, 6 h, 12 h and 24 h) of 1 μM CX-5461 in BJ-T p53sh cells. We compared this response to another inhibitor of Pol I transcription, low dose (5 nM) of Actinomycin D (Act D) at 30 min and 3h (Figure S6A and S6B). After 30 min of CX-5461 treatment only 4 Pol II transcribed genes (*HES1*, *DUSP1*, *MPK-1*, and *CYR61*) associated with early stress response pathways were differentially expressed and no changes in gene expression were detected following 30 min of Act D treatment. Following 1h of CX-5461 treatment, 73 genes were significantly differentially expressed and their gene ontologies as determined by MetaCore^™^ analysis reflect an antiproliferative response as well as DNA damage-ATM/ATR mediated G2-cell cycle checkpoint (Table 1). Further, these ontologies were significantly enriched after 3- and 6-hour treatment with CX-5461 (Table 1, Figure S6C). Differentially expressed genes that were common to all the CX-5461 treatment time points are involved in antiproliferative AP-1 and TGFβ-transcriptional programs as well as immune response pathways including those associated with NF-κB and JAK/STAT signaling pathways (Figure S6C–S6E). These expression signatures including the immune response reflect the senescence phenotype observed following chronic treatment with CX-5461 (Figure S1A) [54]. Gene expression signatures of immune response pathways were also observed after 3 h of Act D treatment (Table 2). Interestingly, the gene expression signature of the DNA damage-ATM/ATR mediated G2 checkpoint was unique to the early treatment time points of CX-5461 (1, 3 and 6 h), consistent with CX-5461-mediated activation of ATM/ATR signaling (Figure 3). Consistent with this Act D treatment (5 nM and 10 nM), which at 3h achieved similar levels of Pol I transcription inhibition compared to CX-5461 did not activate ATM/ATR signaling (Figure 7B), although it induced stabilization of p53 possibly through the canonical ribosomal protein-MDM2 nucleolar stress pathway [28].

**Table 1:** MetaCore ontology analysis of differentially expressed genes identified by RNA-seq following 1-hour and 3-hour treatment of BJ-T p53sh cells with 1 μM CX-5461

Enrichment analysis report						
Enrichment by Pathway Maps			1h CX-5461		3h CX-5461	
#	Maps	Total	*p* - value	In Data	*p* - value	In Data
**1**	Development_WNT signaling pathway. Part 2	**53**	**8.535E-02**	**2**	**1.57188E-09**	**16**
**2**	Reproduction_GnRH signaling	**72**	**1.626E-08**	**9**	**0.00013399**	**12**
**3**	Immune response_HSP60 and HSP70/ TLR signaling pathway	**54**	**1.331E-02**	**3**	**1.82682E-08**	**15**
**4**	Immune response_TLR2 and TLR4 signaling pathways	**57**	**1.820E-03**	**4**	**2.9642E-07**	**14**
**5**	Immune response_IL-18 signaling	**60**	**1.766E-02**	**3**	**5.85884E-07**	**14**
**6**	Immune response_TLR5, TLR7, TLR8 and TLR9 signaling pathways	**48**	**7.349E-05**	**5**	**1.71938E-06**	**12**
**7**	DNA damage_ATM / ATR regulation of G2 / M checkpoint	**26**	**1.678E-03**	**3**	**1.76826E-06**	**9**
**8**	Development_TGF-beta receptor signaling	**50**	**1.114E-03**	**4**	**2.74665E-06**	**12**
**9**	Immune response_C5a signaling	**50**	**7.721E-02**	**2**	**2.74665E-06**	**12**
**10**	Transcription_NF-kB activation pathways	**51**	**7.989E-02**	**2**	**3.43906E-06**	**12**

RNA was extracted from three biological replicates. The most significantly enriched gene ontologies are represented. *p*-value denotes the significance of the number of differentially expressed genes (In Data) compared to the total number of genes (Total) in the gene ontology classification.

**Table:** MetaCore ontology analysis of differentially expressed genes identified by RNA-seq following 3-hour treatment of BJ-T p53sh cells with 5 nM Act D

Enrichment analysis report				
Enrichment by Pathway Maps		3h ActD		
#	Maps	Total	*p* - value	In Data
**1**	Immune response_IL-1 signaling pathway	**44**	**3.048E-08**	**12**
**2**	Apoptosis and survival_APRIL and BAFF signaling	**39**	**7.918E-08**	**11**
**3**	Immune response_TLR2 and TLR4 signaling pathways	**57**	**8.307E-08**	**13**
**4**	Immune response_IL-17 signaling pathways	**60**	**1.589E-07**	**13**
**5**	Signal transduction_NF-kB activation pathways	**51**	**1.826E-07**	**12**
**6**	Immune response_TNF-R2 signaling pathways	**45**	**3.947E-07**	**11**
**7**	Immune response_CD40 signaling	**65**	**4.289E-07**	**13**
**8**	Expression targets of Tissue factor signaling in cancer	**22**	**5.512E-07**	**8**
**9**	Immune response_Signaling pathway mediated by IL-6 and IL-1	**30**	**6.927E-07**	**9**
**10**	Immune response_TLR5, TLR7, TLR8 and TLR9 signaling pathways	**48**	**7.970E-07**	**11**

RNA was extracted from three biological replicates. The most significantly enriched gene ontologies are represented. *p*-value denotes the significance of the number of differentially expressed genes (In Data) compared to the total number of genes (Total) in the gene ontology classification.

At low concentrations, Act D (5 nM) preferentially intercalates into GC-rich regions of rDNA and inhibits Pol I transcription at the level of elongation in contrast to CX-5461, which blocks recruitment of Pol I and pre-initiation complex formation [32, 55, 56]. In agreement with these differential mechanisms of Pol I transcription inhibition, Act D did not affect Pol I recruitment to the rDNA promoter although Pol I binding across the transcribed region was reduced in BJ-T p53sh cells (Figure 7C). In marked contrast, CX-5461 induced a significant reduction in Pol I binding at the rDNA promoter and across the transcribed region (Figure 7C). These observations together with the lack of ATM/ATR activation by Act D are consistent with a model whereby CX-5461-induced defects in Pol I complex assembly at the rDNA promoter rather than rRNA synthesis rates *per se* are responsible for ATM/ATR checkpoint activation.

In human cells, only a subset of the 300 rDNA repeats is transcribed at any given time. Active rDNA chromatin is open/accessible and bound by UBF, which is essential in determining and maintaining the active rDNA state [9, 57–59]. While, silenced rDNA is devoid of UBF and Pol I and can be distinguished from the active rDNA pool by differential accessibility to psoralen followed by Southern blotting [58]. UBF binding to rDNA was not altered in response to CX-5461 or Act D (Figure 7D), nor was the ratio of active to silent rDNA pool affected after 3 h of CX-5461 treatment (Figure S7A). Therefore, upon CX-5461 treatment Pol I is depleted from the rDNA however the rDNA remains stably bound by UBF and in an open configuration structure. Consistent with this, nucleosome positioning by micrcoccal nuclease (MNase) accessibility assay revealed that CX-5461, but not Act D, led to a significant decrease in protection against MNase digestion at the rDNA promoter (Figure 7E), presumably due, at least in part, to loss of Pol I. This suggests that CX-5461-mediated reductions in Pol I recruitment leads to ‘exposed’ rDNA repeats, a configuration that is presumably not encountered during normal physiology. Our data suggest that this abnormal rDNA chromatin leads to activation of the ATM/ATR pathway.

Further, consistent with our previous results [21], CX-5461 treatment is associated with nucleolar disruption as demonstrated by the delocalization of a proportion of NPM1 and Fibrillarin (FBL) into the nucleoplasm (Figure 6D and 6E, Figure S7B) [21]. This correlated with the collapse of the nucleolar organizer regions (NORs) within the nucleoli. CX-5461-mediated condensation of the rDNA loci within the nucleoli is distinct to the reported delocalization of rDNA into the nucleolar periphery following IPpoI-induced rDNA DSBs [47]. This finding further reinforces the notion that the biological response to acute Pol I inhibition by CX-5461 is distinct and independent of DNA damage and that defects in rDNA chromatin or changes in rDNA topology can directly activate ATM/ATR leading to S and G2 checkpoint activation.

## DISCUSSION

We developed a new class of cancer therapeutics that selectively inhibit Pol I transcription [26, 27, 32, 60]. Further, we demonstrated that one of these inhibitors, CX-5461, which is currently undergoing phase 1 clinical trials for haematological cancers, treats lymphomas by activating a nucleolar stress pathway that induces p53-mediated apoptosis [21, 25]. Activation of p53 via ribosomal protein-mediated inactivation of MDM2 is a major mechanism through which cell cycle arrest or apoptosis is induced in response to altered ribosome biogenesis. Importantly however, we also showed that the therapeutic efficacy of CX-5461 to treat solid tumours does not correlate with p53 status [32]. More recently, CX-5461 has been reported to induce a p53-independent G2 arrest and apoptosis dependent upon ATM/ATR activity in lymphoblastic leukaemia [33, 34]. In this paper, we extend these findings by examining the mechanisms underlying the p53-independent cellular response to Pol I transcription inhibition by CX-5461 in order to further improve its application in the clinic. Our studies reveal a p53-independent immediate response to CX-5461 involving rapid activation of G1, S-phase and G2 checkpoints leading to cell cycle arrest, senescence or cell death depending on the cell's genotype. Our data suggest that in the absence of p53, CX-5461-induces a G1 checkpoint that is associated with ATM activation. In addition, CX-5461 induces ATM and ATR-mediated S-phase delay and G2 arrest. Further, we demonstrate that the combination of CX-5461 and inhibition of ATM/ATR signaling in p53-null cells induces mitotic catastrophe and subsequent cell death. Importantly, the combination of CX-5461 and inhibition of ATM/ATR signaling leads to enhanced therapeutic efficacy in treating an aggressive *Tp53*^−/−^ Eμ-*Myc* lymphoma *in vivo*. Inactivation of cell cycle checkpoints leading to mitotic catastrophe is likely to be key to the improved capacity of CX-5461 in killing *Tp53*^−/−^ MYC-driven cancer cells.

CX-5461 was developed as a highly specific inhibitor of Pol I transcription initiation (with ~200-fold higher selectivity for Pol I over Pol II transcription due to its ability to disrupt the recruitment of the selectivity factor 1 (SL-1) to the rDNA promoter [32]. Unlike quarfloxin (CX-3543), which is a G-quadruplex (G4) interactive agent that inhibits Pol I by disrupting nucleolin/rDNA G4 complexes [60], CX-5461 was not developed to target G4 DNA. Consistent with this, we do not detect G-quadruplex stabilization with CX-5461 at 1 μM for 1 h in BJ-T cells using the 1H6 antibody [61], which is specific to different G4 DNA structures (results not shown). This suggests that of G-quadruplex stabilization, which had been associated with replication defects and subsequent activation of ATR mediated-ATM signaling [62], is unlikely to account for the induction of ATM and ATR signaling by CX-5461. On the other hand, Ellipticines interact with DNA and specifically inhibits Pol I transcription by affecting the three-dimensional rDNA structure and preventing SL-1 from binding to the rRNA promoter. However, Ellipticines induced ATM/ATR-independent cell cycle defects [63]. To further test the specificity of CX-5461 in activating ATM/ATR pathway signaling, we utilized CX-5447, a closely related structural analogue of CX-5461 that is inactive against Pol I transcription [32], does not induce CHK2 (T68) phosphorylation (Figure S8A and S8B) and has no effect on cell cycle progression (Figure S8C). Therefore, our data strongly suggest that CX-5461′s ability to rapidly block Pol I recruitment and binding to the rDNA leading to exposed rDNA structures, triggers ATM/ATR pathway activation as opposed to off-target effects of the drug unrelated to Pol I inhibition. In an attempt to further demonstrate the specificity of inhibiting Pol I transcription initiation in activating ATM/ATR pathway signaling, we performed RNA interference (RNAi)-mediated knockdown of POLR1A, the largest subunit of the Pol I complex and the Pol I specific transcription initiation factor RRN3 in order to inhibit Pol I transcription initiation by means other than CX-5461 (Figure S8D). However, knocking down POLR1A and RRN3 protein levels by greater than 90% after 48 hours of siRNA transfection only reduced 47S rRNA precursor levels by 25% and 50% compared to control after 12 hours and 48 hours of transfection, respectively (Figure S8D and S8E). siRNA knock down of POLR1A and RRN3 was associated with the induction of p53 protein levels and G2 cell cycle arrest but it did not lead to activation of ATM/ATR signaling (Figure S8B and S8C). By comparison, CX-5461 caused acute reduction in 47S rRNA levels by 70% within 30 min that was maintained at this level or greater over 48 hours (Figure S8F and Figure7A). The temporal and quantitative differences in cellular responses to these two approaches of inhibiting Pol I transcription initiation is consistent with previous studies demonstrating that RNAi phenotypes do not always correlate with small molecule inhibition profiles for a number of biological reasons [64]. In particular, the inability of RNAi targeting Pol I to robustly inhibit Pol I activity may be due to the fact that less than 10% of the total Pol I enzyme complex is transcriptionally active at any one time [65]. Thus, even removing 90% of the available Pol I enzyme may have little effect on the pool of active polymerase. Moreover, effective silencing of gene expression using RNAi is in part dependent on the half-life of the targeted protein and occurs on the timescale of hours to days after transfection providing ample time for cellular compensatory responses to be activated. In contrast, chemical approaches immediately inhibit their target leaving little opportunity for activation of compensatory mechanisms. Taken together, our data support a model by which CX-5461 rapidly inhibits Pol I loading on the rDNA repeat leading to exposed rDNA chromatin that activates ATM signaling within the nucleoli. Such rapid effects on rDNA topology cannot be temporally replicated through RNAi to Pol I components.

The rapid (within 30 min) activation of ATM/ATR signaling by CX-5461 occurs in the absence of global DNA damage (Figure 3 and Figure 6). This is consistent with reports suggesting that DDR may differ considerably depending on alternative triggers, sensing mechanisms and cellular context [51]. For instance, oxidative stress-mediated non-canonical activation of ATM occurs in the absence of DSBs and does not lead to formation of γH2AX domains. Yet, CHK2 and p53 are phosphorylated by oxidation-activated ATM [50]. We did not observe γH2AX foci formation nor induction in γH2AX protein levels following treatment with CX-5461 over short time points (up to 6 h) (Figure 6B). However, we have observed γH2AX foci formation following long-term treatment with CX-5461 of BJ-T cells (greater than 48 h) (Figure S9A), which did not coincide with either the CX-5461-mediated rapid reduction in rDNA transcription or alterations in nucleolar structure. Instead, it coincided with increased β-galactosidase staining (Figure S1A) suggesting that the formation γH2AX foci is a delayed and indirect response to treatment with CX-5461. Importantly, the γH2AX signal did not overlap with rDNA implying that DSBs did not arise at the rDNA (Figure S9A). Thus, this is consistent with CX-5461′s acute activation of ATM/ATR without DNA damage. Interestingly, pre-treatment of BJ-T cells with CX-5461 prior to exposure to UV radiation led to an increase in UV-induced DNA damage as determined by increased length of comet tails (Figure S9B), suggesting that CX-5461 treatment increases cells sensitivity to DNA damaging agents. This could be due to attenuated DNA repair as a consequence of CX-5461-mediated deregulation of nucleolar structure and function [66]. Importantly, this raises the possibility of enhanced efficacy for CX-5461 in treating cancer cells with compromised DNA repair pathways as well as the potential of combining CX-5461 with standard chemotherapy to improve therapeutic efficacy.

Furthermore, tumours bearing high levels of oncogene-induced DNA damage response (DDR), such as *MYC* driven murine lymphoma (Eμ-*Myc*) and human B-cell lymphomas, are sensitive to therapeutic targeting of DNA-dependent Protein Kinase (DNA-PK) and ATM/ ATR [67–69]. Inhibition of aberrantly active DDR signaling pathway has become an attractive therapeutic strategy in cancer therapy with highly selective small molecule inhibitors of ATM and ATR signaling in preclinical and clinical development, respectively [70]. Thus, the potential efficacy of combining DDR pathway inhibitors with CX-5461 provides a rationale for further clinical evaluation of this therapeutic strategy particularly in p53-null cancers.

Mechanistically, our study strongly suggests that CX-5461 induces chromatin changes at the rDNA repeats leading to activation of ATM signaling within the nucleoli. Indeed, a role for chromatin structure and dynamics as triggers for ATM/ATR activation in the absence of DNA breaks is consistent with several other reports [71–74]. Treatments that perturb chromatin structure without apparently inducing DNA damage, such as hypotonic conditions, chloroquine, trichostatin A, chromatin factor depletion and spliceosome displacement have been reported to induce ATM kinase activity [51, 52, 73, 75–77]. Therefore, our study suggests that CX-5461-mediated perturbations in rDNA chromatin such as, “exposed” structures devoid of Pol I and alterations in rDNA topology can directly activate ATM/ATR. Taken together, our data demonstrate that the integrity of rDNA chromatin is directly coupled to ATM/ATR signaling.

In summary, our data identify a novel p53-independent nucleolar stress pathway that senses perturbations in rDNA chromatin structure and dynamics leading to activation of ATM/ATR signaling and a prompt halt in cell cycle progression and provide a rationale for clinical evaluation of combining DDR pathway inhibitors with CX-5461 to treat in p53-null cancers.

## MATERIALS AND METHODS

### Cell culture, retroviral infection and treatment with pharmacological inhibitors

BJ cell lines were cultured in DMEM with 10% FBS (fetal bovine serum) at 37°C in 5% CO_2_ atmosphere. BJ-T shRNA stable cell lines were generated using retroviral transduction with pRetroSuper (pRS) vectors, which confers puromycin resistance, kindly provided by R. Agami and R. Bernards (The Netherlands Cancer Institute) [78]. The shRNA targeting p53 was generated using the following 19-nucleotide sequences (5′-GACTCCAGTGGTAATCTAC-3′) that act as short interfering RNA (siRNA)-like molecules to stably suppress gene expression [78]. The FUCCI-labeled BJ-T p53sh cell line was produced by lentiviral transduction of pCSII-EF-mCherry-hCdt1(30/120) and pCSII-EF-mVenus-hGeminin(1/110) (kindly provided by Dr. Atsushi Miyawaki, RIKEN, Japan) [49]. Eμ-*Myc* clonal B-lymphoma cell lines were generated and cultured as described previously [21, 69].

CX-5461 and CX-5447 were provided by Cylene Pharmaceuticals and Senhwa Biosciences (San Diego, CA, USA). For use *in vitro* 10mM stocks of CX-5461 and CX-5447 in 50 mM NaH_2_PO_4_ (Vehicle), 10 mM stocks of KU-55933, VE-821 and AZD7762 (obtained from Selleckchem) were prepared in DMSO and 1 mM stocks of Acintomycin D (Act D) (Sigma) were prepared in ethanol.

### Cell cycle analysis

For cell cycle analysis experiments using propidium iodide (PI), cells were pelleted and fixed in 80% ice-cold ethanol and stored at 4°C until further processing. Cells were stained with PI at 50 μg/ml in PBS containing RNase A and analyzed by flow cytometry on the BD FACSCanto II analyzer (BD Biosciences). The percentage of cells in G0/G1, S and G2/M phases were determined using Modfit 3.0 software.

For cell cycle analysis using 5-bromo-2′-deoxyuridine (BrdU) incorporation, cells were labeled with 10 μM BrdU for 30 min, washed twice with PBS, collected and harvested as above. Cells were pelleted and incubated in 1 mL of 2N HCl containing 0.5% (v/v) Triton X-100 for 30 min then pelleted and washed in 1 mL of 0.1M Na_2_B_4_O_7_.10H_2_O (pH 8.5). Cell pellets were sequentially incubated for 30 min with anti-BrdU and Alexa Fluor 488 anti-mouse IgG antibodies (Supplemental methods, Table 2) in PBS containing 2% FBS and 0.5% Tween-20. Cells were washed with PBS- 2% FBS then incubated in RNaseA containing 10 mg/mL PI solution at 37°C for 15 min. Cells were analyzed on the FACSCanto II and cell-cycle analysis was performed using FCS Express software (De Novo, Los Angeles, CA, USA).

For Annexin-V analysis cells were stained with Annexin-V-APC (BD Pharmigen 550474) and 10 μg/mL PI and analyzed with the BD FACSCanto II. Flow cytometry data was analysed with FCSExpress software.

### Animal experiments

All animal experiments were performed with approval from the Peter MacCallum Cancer Centre Animal Experimentation Ethics Committee. C57Bl/6 mice (Walter and Eliza Hall Institute, Parkville, VIC, Australia) were intravenously injected with 2 × 10^**5**^ Eμ-*Myc* B-lymphoma cells in PBS and treated with pharmacological inhibitors from 8 days post-injection. Treatment of mice was continued until an ethical end-point was reached; hunched posture, ruffled fur, enlarged lymph nodes, laboured breathing, weight loss greater than 20% of start body weight and limited mobility or paralysis.

For use *in vivo* CX-5461 was prepared in 25 mM NaH_2_PO_4_ (pH4.5) and given by oral gavage at 30 mg/kg every three days. AZD7762 (Medchem Express) was delivered intraperitoneally in 10.3% -hydroxypropyl-β-cyclodextrin in 0.9% saline at 20 mg/kg daily on weekdays.

Reverse transcription qPCR, Western blot analysis, ChIP, Immunofluorescence-fluorescent *in situ* hybridisation (IF-FISH), psoralen crosslinking and chromatin accessibility by real time-PCR (CHART-PCR) assays were carried out as described previously [58, 59]. A brief summary of these assays and lists of antibodies and primer sequences are provided in the Supplementary Data.

### Comet assay

Comet Assays were performed under alkaline conditions using Trevigen CometAssay Reagent Kit (Cat. # 4250-050-K).

### RNA-seq

Sequencing libraries were prepared using the TruSeq RNA sample preparation kit (Illumina) and sequenced on a Illumina HiSeq2500 platform at Peter MacCallum Cancer Centre (50 bp, PE). The generated 50 bp paired-end reads were aligned to the genome using Bowtie2 [79] with default parameters and the reads counted using HTSeq [80]. The differential expression was then calculated utilizing the DEseq package [81] in R (version 3.0.2) [82]. Only differentially expressed genes with FDR ≤ 0.1 and logFC ≤ −0.5 or logFC ≥ 0.5 were considered for further analysis.

### Statistical analysis

was performed using Student's paired *t*-tests. The significance of difference in survival curves (Figure 5) was performed using Log-rank (Mantel-Cox) test.

## SUPPLEMENTARY MATERIALS FIGURES AND TABLES


