# Supplementary material for: Inhibition of RNA polymerase I transcription initiation by CX-5461 activates non-canonical ATM/ATR signaling
Source: Oncotarget. 2016 Jul 6;7(31):49800–18. doi: 10.18632/oncotarget.10452 (PMC5226549; doi:10.18632/oncotarget.10452)
Supplement: Supplementary file 1 [file oncotarget-07-49800-s001.pdf]

Inhibition of RNA polymerase I transcription initiation by CX-5461 activates non-canonical ATM/ATR signaling

Supplementary Materials

siRNA knockdown and RNA expression analysis

Dharmafect 1 reagent (Dharmacon) was used to transfect siRNA at 40 nM. Cells were lysed, RNA was extracted, and first-strand cDNA was synthesized using random hexamer primers and Superscript III (Invitrogen). Quantitative PCR (qPCR) was performed in duplicate using the FAST SYBR Green dye on the StepOnePlus real- time PCR system (Applied Biosystems). Primer sequences are listed in (Supplementary Table 1).

siEGFP: sense 5′-GCAGCACGACTTCTTCAA G-3′, siEGFP anti-sense 5′CTTGAAGAAGTCGTGCTG C-3′ oligos were synthesized by Sigma.

siGenome SMARTpool siRNA for siPOLR1A (M-013983) and siRRN3 (M-016947) were obtained from Dharmacon.

Cell cycle analysis of mitotic cells

For analysis of mitotic cells by phospho-H3 staining, cells were collected, washed in PBS, fixed in ice-cold 90% ethanol, and incubated at 4°C. Cells were resuspened in PBS + 0.25% Triton X-100 and incubated sequentially with anti-phospho-H3(Ser10) and Alexa Fluor 488 donkey anti-rabbit IgG (Table 2) at room temperature. Cell were washed and resuspended in 10 μg/ml propidium iodide (PI) and analysed by flow cytometry on a BD FACS Canto II. Quantitation of cell cycle populations were performed using FCS Express and Mod Fit 3.0 analysis software.

The U2TR cell line

The U2OS cells expressing U2TR IPpoI-dd (U2TR) were provided by Prof. Kum Kum Khanna (The University of Queensland, Australia). The tetracyclin inducible U2TR IPpoI -dd system allows expression of IPpoI-destabilization domain (dd) fusion protein, which is targeted for proteasomal degradation. Shield-1 ligand binds to the dd and stabilizes the IPpoI -dd protein [1]. IPpoI endonuclease cuts DNA with high specificity at its target sequence within the 28S region of rDNA, resulting in DSBs.

Sequences of qPCR primer flanking IPpoI rDNA cut site [2]

Forward-5′ACGCGATGTGATTTCTGCCC3′

Reverse- 5′TCTTCTTTCCCCGCTGATTCC3′

Western blotting (WB)

Twenty to fifty milligrams of whole-cell lysates were resolved by SDS- PAGE, electrophoretically transferred onto PVDF membranes (Milli- pore), and analyzed using enhanced chemiluminescence (ECL) detection (GE Healthcare). Antibodies details are listed in Supplementary Table 2

IF-FISH

Cells were fixed in 4% paraformaldehyde (10 min at room temperature), washed with PBS, and blocked with 5% skim milk powder, 0.5% chicken serum in PBS, and 0.3% Triton ×-100 for 30 min. Cells were sequentially incubated with the primary antibody and secondary antibodies (Supplementary Table 2). Stained cells were fixed again with 4% formaldehyde and counterstained in Vectashield mounting media (Vector Labs).

Following performing immunofluorescence, slides were fixed in methanol:acetic acid (3:1) for 5 min at room temperature then dried dehydrated through 70%–80% ethanol series. Slideswere denatured in 70% formamide/2XSSC for 10 min at 83°C and then dehydrated through ethanol series and air-dried. Probes derived from either the intergenic spacer of the human ribosomal gene repeat (kindly provided by Prof. Brain McStay, NUI Galway). 100 ng of denatured biotin-labeled probe were combined with 30 μg salmon sperm DNA and 18 μg Cot1 carrier DNA (Invitrogen) in (50% formamide, 20% dextran sulphate in 2XSSC) were added per slide to hybridize at 37°C for 24 h in a humidified chamber. Slides were washed in 50% formamide/2XSSC at 42°C for 15 min and 0.1XSSC at 60°C for 15 min. Streptavidin-Alexafluor 488 was added for 1hr at 37°C and slides were then washed in 0.05% Tween-20/4XSSC for 15 min. Slides were mounted in DAPI-Vectashield mounting media (Vector Labs). Images were acquired on an Olympus BX-51 microscope equipped with a Spot RT camera (model 25.4), using the UPlanAPO 60X, NA 1.2 water immersion objective and the Spot Advanced software, version 4.6.4.3. Settings for adjusting the image after acquisition (i.e. gamma adjust and background subtract settings) were identical for all images.

ChIP

ChIP was performed as described previously [3]. Crosslinking was achieved with 0.6% formaldehyde. Chromatin was sheared using Covaris and assays performed using 2 × 10^6 cells per immunoprecipitation. For all ChIPs, 4 µg of purified antibody or 8 µl of sera was used per immunoprecipitation. Samples were analyzed in triplicate using the SYBR green dye on the ABI Prism 7000 (Applied Biosystems). To calculate the percentage of total DNA bound, unprecipitated input samples from each condition were used as reference for all qRT-PCR reactions. Primer sequences are listed in Supplementary Table 3

Psoralen crosslinking assay

Cells were lysed in 10 mM Tris-HCl, pH 7.4, 10 mM NaCl, 3 mM $MgCl_2$, and 0.5% NP-40, and nuclei were pelleted, resuspended in 50 mM Tris- HCl, pH 8.3, 40% glycerol, 5 mM $MgCl_2$, and 0.1 mM EDTA, and irradiated in the presence of 4,5,8′-trimethylpsoralen (Sigma-Aldrich) with a 366-nm UV light box at a distance of 6 cm. 200 µg/ml psoralen was added at 1:20 dilution every 4 min for a total irradiation time of 20 min. Genomic DNA was isolated, digested with SalI, and separated on a 0.9% agarose gel, and alkaline Southern blotting was performed. To reverse psoralen crosslinking, filters were treated with 254-nm UV rays at 1,875 × 100 µJ/cm^2 using a UV cross-linker (Stratalinker 2400; Agilent Technologies). The membrane was then hybridized to a purified ^{32}P (Amersham)-labeled rDNA fragment (+1601 − +2089nt) targeting the 5′external transcribed spacer, visualized by scanning on a PhosphoImager (GE Healthcare), and quantitated using ImageQuant (TLv2005.04; GE Healthcare).

CHART-PCR

Cells were collected and mononucleosome preparations were performed using an EpiScope nucleosome preparation kit (#5333; Takara). Briefly, nuclei were isolated from control, CX-5461 and Act D treated cells and incubated at 37°C for 30 min with MNase. A control without MNase was included for each sample. gDNA was then isolated and subjected to qPCR using FAST SYBR Green dye on the StepOnePlus real-time PCR system (Applied Biosystems). The relative level of MNase resistance was calculated after normalisation to mock-digested DNA.

Β-galactosidase staining of senescent cells was performed as described in [4, 5].

REFERENCES

1. Banaszynski LA, Chen LC, Maynard-Smith LA, Ooi AG, Wandless TJ. A rapid, reversible, and tunable method to regulate protein function in living cells using synthetic small molecules. Cell 2006; 126:995–1004.
2. Berkovich E, Monnat RJ Jr., Kastan MB. Roles of ATM and NBS1 in chromatin structure modulation and DNA double-strand break repair. Nat Cell Biol. 2007; 9:683–690.
3. Poortinga G, Hannan KM, Snelling H, Walkley CR, Jenkins A, Sharkey K, Wall M, Brandenburger Y, Palatsides M, Pearson RB, McArthur GA, Hannan RD. MAD1 and c-MYC regulate UBF and rDNA transcription during granulocyte differentiation. The EMBO journal. 2004; 23:3325–3335.
4. Itahana K, Campisi J, Dimri GP. Methods to detect biomarkers of cellular senescence: the senescence-associated beta-galactosidase assay. Methods Mol Biol. 2007; 371:21–31.
5. Astle MV, Hannan KM, Ng PY, Lee RS, George AJ, Hsu AK, Haupt Y, Hannan RD, Pearson RB. AKT induces senescence in human cells via mTORC1 and p53 in the absence of DNA damage: implications for targeting mTOR during malignancy. Oncogene. 2012; 31:1949–1962.

Supplementary Table S1: qPCR primer sequences for reverse transcription-RT-PCR

	Forward	Reverse
47S-5'ETS	GGCGGTTTGAGTGAGACGAGA	ACGTGCGCTCACCGAGAGCAG
MYC	GGACGACGAGACCTTCATCAA	CCAGCTTCTCTGAGACGAGCTT
Vimentin	AGAGAACTTTGCCGTTGAAGCT	GAAGGTGACGAGCCATTTCC

Supplementary Table S2: Antibodies

Antibody	Company	Catalogue No.	Application
p53 (DO-1)	Santa Cruz	sc-126	WB
pp53	Cell Signaling	9284	WB
p21(C-19)	Santa Cruz	sc-397	WB
Tubulin	Sigma	C-4585	WB
pCHK1 (S345) (133D3)	Cell Signaling	2348	WB
CHK1(FL-476)	Santa Cruz	SC-7898	WB
pCHK2 (T68) (C13C1)	Cell signaling	2197	WB
CHK2	Cell signaling	2662	WB
H2AX	Abcam	ab20669	WB
γH2AX	Millipore	05-636-I	WB, IF
γH2AX	Abcam	ab81299	IF in FUCCI labeled cells
CDK1 [Cdc2 p34 (H-297)]	Santa Cruz	sc-747	WB
pCDK1 (Y15)	Cell signaling	2348	WB
Active RB	Bd Pharmingen	554164	WB
Cyclin B (H433)	Santa Cruz	sc-752	WB
pATM (S1981) [EP1890Y]	Abcam	ab81292	WB
ATM Clone 2C1	GeneTex	ab81292	WB
Actin	MP Biosciences	691002	WB
POLR1A (RPA194)	Santa Cruz	sc-48385	WB
pNBS1 (S343) (Clone EP178)	GeneTex	GTX61779	IF
Goat-α-Mouse HRP	BioRAD	172-1011	WB
Goat-α-Sheep HRP	Dako	P016302-2	WB
Goat-α-Rabbit HRP	BioRAD	170-6515	WB
Donkey anti-mouse IgG- Alexa Fluor 488	Invitrogen	A21202	IF, FACS
Donkey anti-mouse IgG-Alexa Fluor 594	Invitrogen	A21203	IF
Goat anti-mouse IgG-Alexa Fluor 405	Abcam	Ab175660	IF in FUCCI labeled cells
Donkey anti-rabbit IgG-Alexa Fluor 647	Abcam	Ab150079	IF in FUCCI labeled cells
Anti-bromodeoxyuridine	Roche	1170376	FACS
Phospho-H3(Ser10)	Millipore	06-570	FACS
AnnexinV-APC	BD Pharmigen	550475	FACS

Anti-UBF, Pol I (POLR1A) and rabbit sera antibodies used for ChIP were generated in-house. The anti-RRN3 antibody was kindly provided by Prof. Brain McStay, NUI Galway.

Supplementary Table S3: qPCR primer sequences for ChIP

	Forward 5′→3′	Reverse 5′→3′	Location GeBank: U13369.1
ENH	AGAGGGGCTGCGTTTTCGGCC	CGAGACAGATCCGGCTGGCAG	41982-42075
Promoter	CCCGGGGGAGGTATATCTTT	CCAACCTCTCCGACGACA	42943-43
ETS1	GCTCTTCGATCGATGTGGTGACG	CGGGCGGAGCGAGAAGGAC	413-521
ETS2	GGCGGTTTGAGTGAGACGAGA	ACGTGCGCTCACCGAGAGCAG	952-1030
18S	CGACGACCCATTCGAACGTCT	CTCTCCGGAATCGAACCCTGA	3990-4092
ITS1	GAAACCTTCCGACCCCTCT	GCCAGACGAGACAGCAAAC	6455-6507
ITS2	GAGAGAGACGGGGAGGGCGG	CCGAGGGAGGAACCCGGA CC	6995-7104
28S	AGTCGGGTTGCTTGGGAATGC	CCCTTACGGTACTTGTTGACT	8204-8300
Terminator	ACCTGGCGCTAAACCATTCGT	GGACAAACCCTTGTGTCGAGG	12855-12970
RFB	GTGTAGGAGTGCCCGTCG	AAATGTGGGAGAGGGAGTTC	13534-13790
IGS	GTTGACGTACAGGGTGGACTG	GGAAGTTGTCTTCACGCCTGA	18155-18280

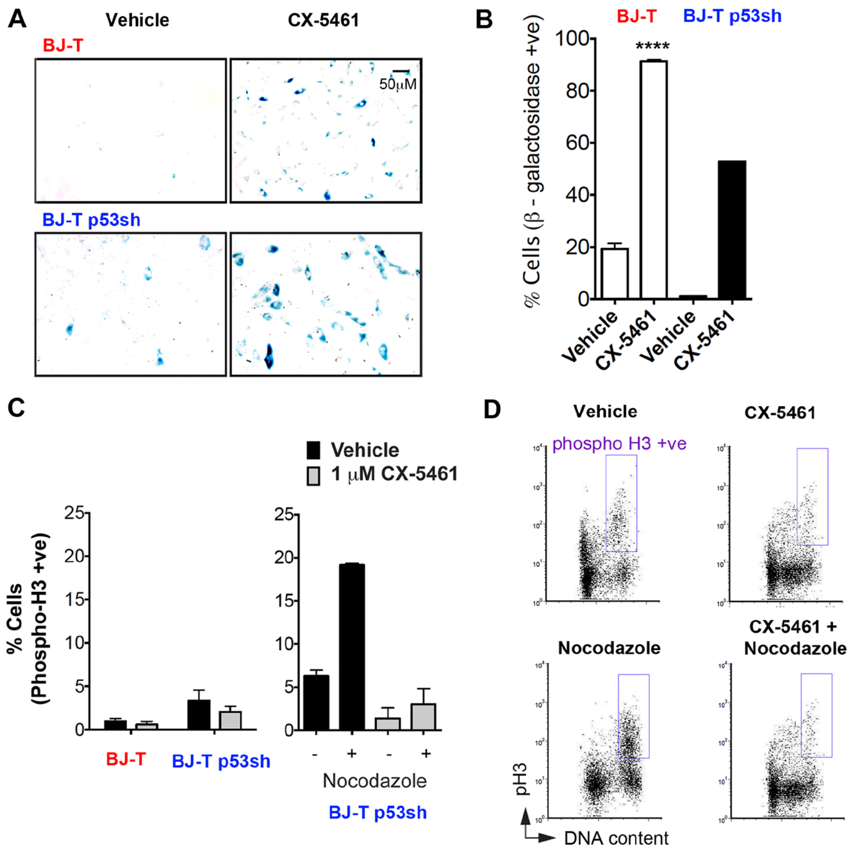

Supplementary Figure S1: Inhibition of Pol I transcription initiation by CX-5461 induces cell cycle arrest and senescence independently of p53. (**A**) β-galactosidase staining BJ-T and BJ-T p53sh cells treated 1 μM CX-5461 with 3 and 7 days, respectively. (**B**) Quantitation of senescence associated β-galactosidase positive cells as percentage of total cells of in BJ-T ($n = 4$) and BJ-T p53sh ($n = 1$) as in (A). Error bars represent mean ± s.e.m, ****p-value < 0.0001. (**C**) Phospho-Histone H3 staining analysis of mitotic cells in BJ-T and BJ-T p53shRNA cell lines treated with 1 μM CX-5461 for 24 h (left panel) ($n = 4$), error bars represent mean ± s.e.m. BJ-T p53sh cells were arrested in mitosis using 50 μM nocodazole for 24 h in the presence or absence of 1 μM CX-5461 (right panel). No significant increases in % of phospho-H3 (S10) positive cells following CX-5461 treatment were detected suggesting that the cells arrest in G2 before reaching mitosis ($n = 2$), error bars represent mean ± s.e.m. (**D**) Analytical FACS analysis showing phospho-H3 (S10) staining as a function of DNA content of BJ-T p53sh cells treated with vehicle or 50 μM nocodazole for 24 h in the presence or absence of 1 μM CX-5461.

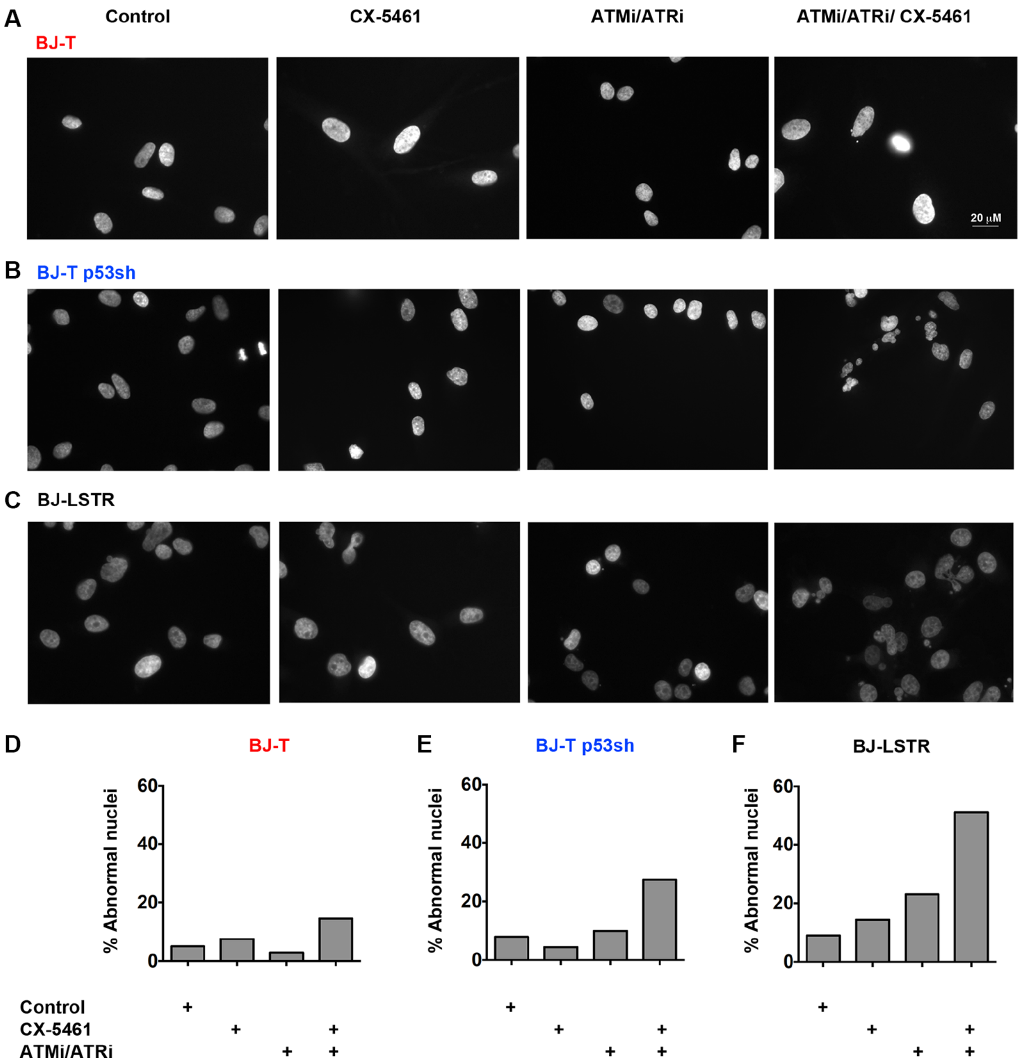

Supplementary Figure S2: Combination treatment of ATM and ATR inhibitors with CX-5461 induces mitotic catastrophe in BJ-T p53sh and BJ-LSTR cells. DAPI-stained BJ-T (**A**), BJ-T p53sh (**B**) and BJ-LSTR cells (**C**) following 24 h treatment with vehicle or 1 µM CX-5461 in combination with DMSO (control) or 5 µM ATMi/5 µM ATRi. Mitotic catastrophe is characterized by appearance of fragmented abnormal nuclei and the presence of micronuclei. (**D**) Quantitation of the percentage of abnormal nuclei. Approximately 200 nuclei for each treatment were counted.

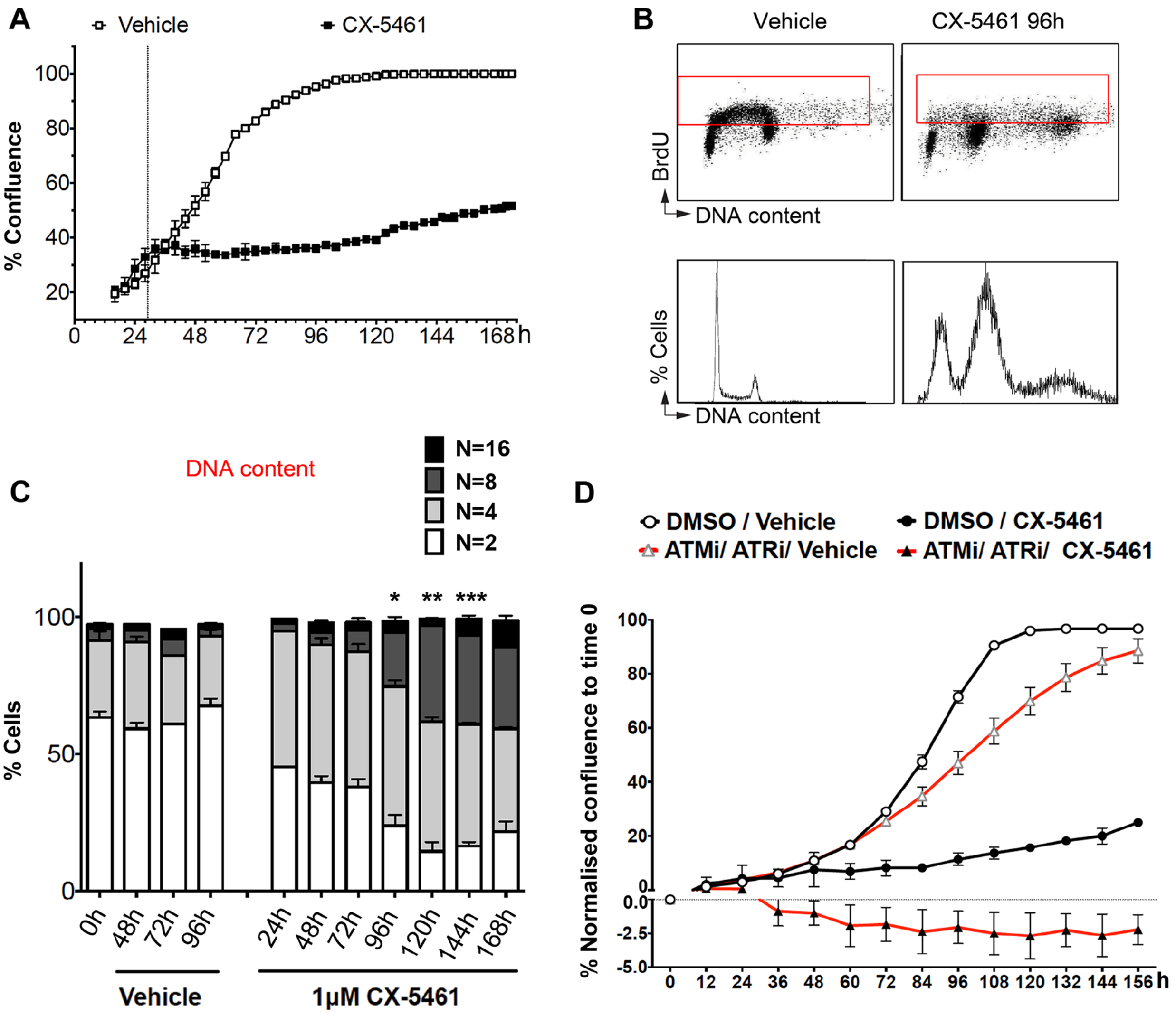

Supplementary Figure S3: CX-5461 treatment of the BJ-LSTR cells causes a defect in cell proliferation and cell death when combined with ATM and ATR inhibitors. (**A**) Proliferation time course using IncuCyte ZOOM of the BJ-T cell lines. Dashed line indicates the addition of vehicle or 1 μM CX-5461. Error bars represent mean ± s.d. of 2 technical replicates (representative of $n = 6$). (**B**) Cell cycle analysis of BJ-LSTR treated with Vehicle or 1 μM CX-5461 for 96 h. BrdU incorporation analysis (upper panels) and PI staining for DNA content (lower panels) were performed as described in Figure 2A (representative of $n = 3$). (**C**) Cell cycle analysis of BJ-LSTR cells treated with either vehicle or 1 μM CX-5461 over the indicated time course. Quantitation of percentages of cells based on PI staining for DNA content $N = 2$ (G0/G1), $N < 4$ (S) and $N = 4$ (G2/M), $N = 8$ and $N = 16$ were determined using FCS express software ($n = 5$), error bars represent mean ± s.e.m, *$p < 0.05$, **p-value < 0.01, ***p-value < 0.001. (**D**) Proliferation time course using IncuCyte ZOOM of BJ-LSTR cells treated with vehicle or 100 nM CX-5461 in the presence of DMSO or (5 μM ATMi/ 5 μM ATRi). Confluency values was normalised to percentage confluency at time 0 h ($n = 3$), error bars represent mean ± s.e.m.

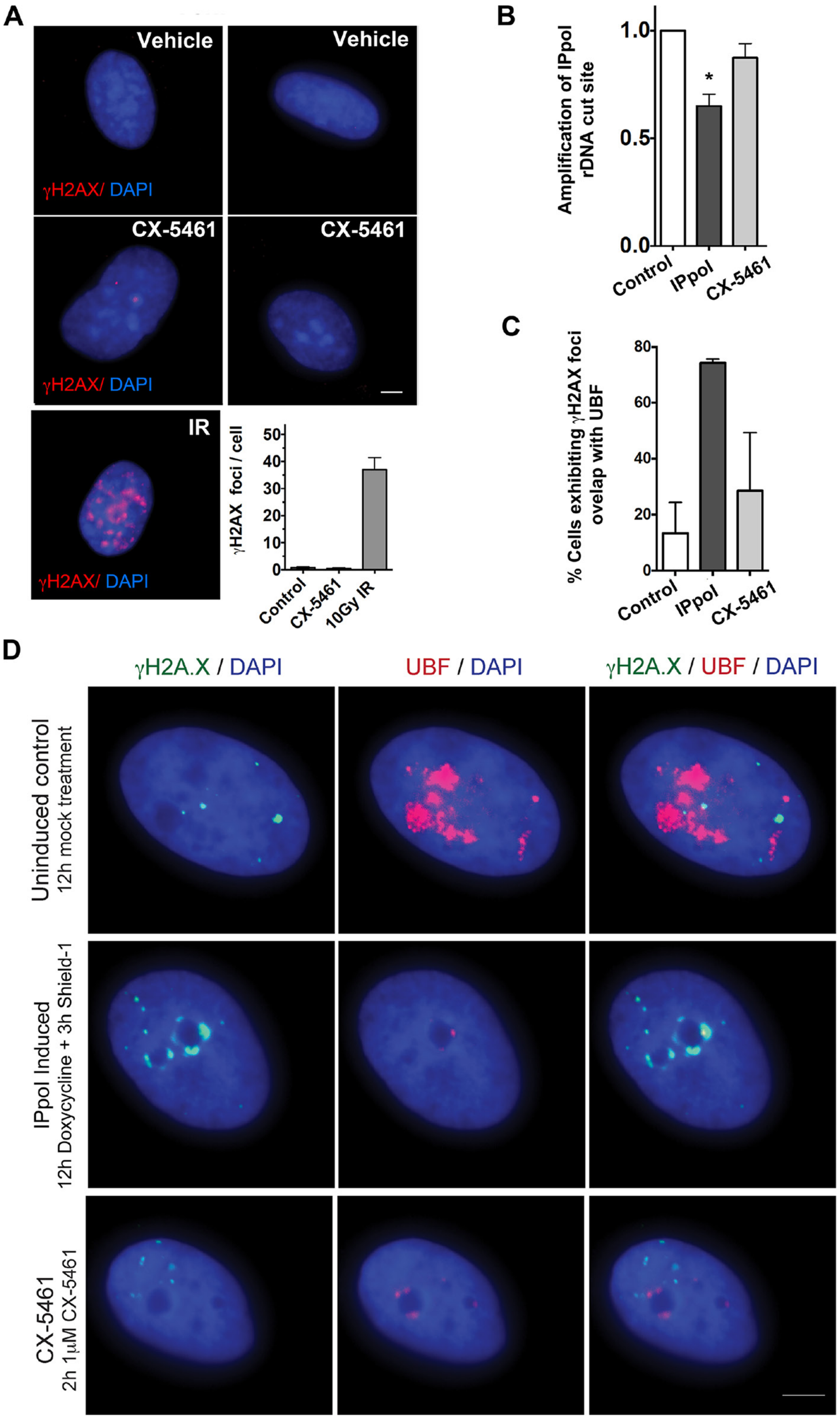

Supplementary Figure S4: CX-5461 does not induce global DNA damage. (**A**) BJ-T cells were treated with vehicle, 1 μM CX-5461 ($n = 4$) or IR 10 Gy ($n = 2$) for 30 minutes and analysed by immunofluorescence (IF) for γH2A.X. Scale bar = 5 μM. Error bars represent mean ± s.d. (**B**) Introduction of DSBs into rDNA using inducible IPpoI endonuclease. The tetracyclin inducible U2TR IPpoI-dd system allows expression of IPpoI- destabilization domain (dd) fusion protein, which is targeted for proteasomal degradation. Shield-1 ligand binds to the dd and stabilizes the IPpoI-dd protein. U2OS cells expressing U2TR IPpoI-dd (U2TR) were incubated in the presence of 1 μg/ml Doxycycline for 12 h (control) followed by 3-hour treatment with the shield-1 ligand to induce IPpoI stabilization in the presence or absence of 1 μM CX-5461. QPCR analysis of levels of DSBs at the IPpoI target site within the 28S region of rDNA. Data presented as fold change in amplification across the IPpoI cut site relative to the rDNA promoter ($n = 3$), error bars represent mean ± s.e.m $*p < 0.05$, $**p < 0.01$. (**C–D**) co-IF for γH2A.X and UBF, a nucleolar marker of cells treated as in (B). Scale bar = 5 μM. The graphs represent quantitation of U2TR cells containing γH2A.X foci associated with the nucleoli following IPpoI induction or CX-5461 treatment ($n = 2$), error bars represent mean ± s.d.

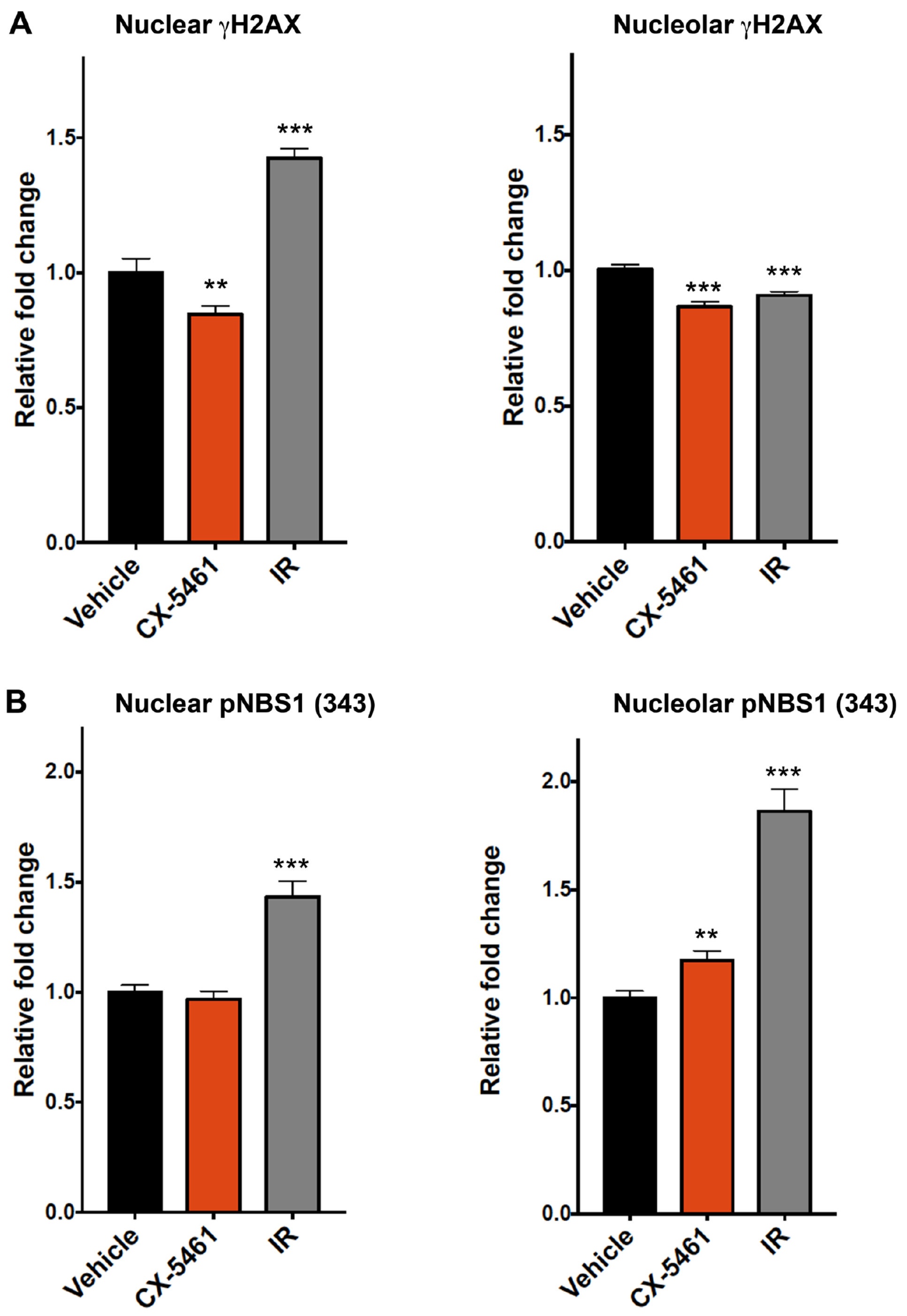

Supplementary Figure S5: CX-5461 activates ATM signaling within the nucleoli in the absence of DNA damage. Quantitation of γH2AX (**A**) and pNBS1 (S343) (**B**) signal intensities in the S and G2 populations in FUCCI-labelled BJ-T p53sh treated with vehicle, 1 µM CX-5461 or 10 Gy IR for 1 h. FUCCI cells express a fragment of Cdt1 linked to the fluorescent protein mCherry during the G1 phase of the cell cycle, as well as a fragment of Geminin linked to the fluorescent protein Venus during the S/G2/M stages. The analysis was performed using the automated Definiens imaging software. The S and G2 populations were selected based on signal ratio of G1/G2 < 1.2. The mean nuclear signal (left panel) or nucleolar signal that overlapped with NPM1 staining (right panel) were normalised to the average signal intensity of the corresponding vehicle controls. Error bars represent mean ± s.d, ** $p < 0.01$, *** $p < 0.001$. Over 200 nuclei per condition were examined from two independent experiments.

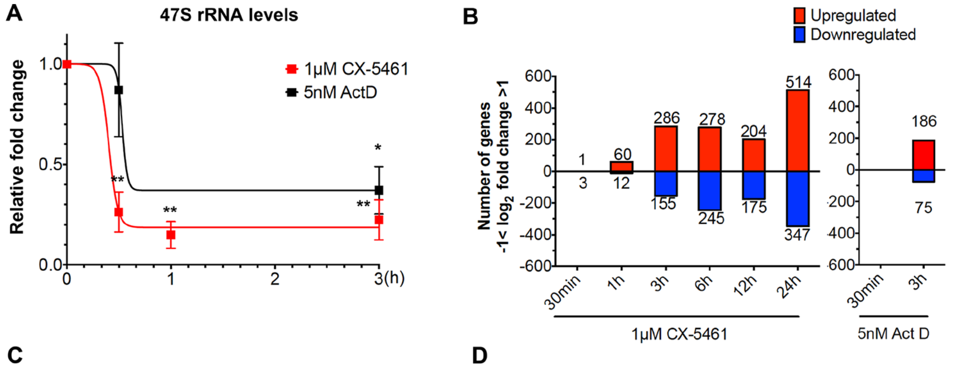

C

Enrichment analysis report				
Enrichment by Pathway Maps		CX-5461, 6 hours		
#	Maps	Total	p-value	In Data
1	Reproduction_GnRH signaling	72	5.553E-09	20
2	Development_PEDF signaling	49	1.475E-08	16
3	Immune response_C5a signaling	50	1.498E-07	15
4	Development_Role of HDAC and calcium/calmodulin-dep	54	4.588E-07	15
5	NF-AT signaling in cardiac hypertrophy	65	1.146E-06	16
6	DNA damage_ATM / ATR regulation of G2 / M checkpoint	26	1.413E-06	10
7	Cell cycle_Role of Nek in cell cycle regulation	32	1.543E-06	11
8	Immune response_TNF-R2 signaling pathways	45	1.650E-06	13
9	Immune response_IL-17 signaling pathways	60	2.005E-06	15
10	Immune response_IL-18 signaling	60	2.005E-06	15

D

Enrichment analysis report				
Enrichment by Pathway Maps		CX-5461, 12 hours		
#	Maps	Total	p-value	In Data
1	Colorectal cancer (general schema)	30	6.240E-08	11
2	Reproduction_GnRH signaling	72	1.943E-07	16
3	Development_TGF-beta-dependent induction of EMT via MAPK	47	1.378E-06	12
4	Development_Regulation of epithelial-to-mesenchymal transition	64	1.392E-06	14
5	Breast cancer (general schema)	41	2.239E-06	11
6	Transcription_Role of AP-1 in regulation of cellular metabolism	38	7.886E-06	10
7	Mitogenic action of Estradiol / ESR1 (nuclear) in breast cancer	28	3.389E-05	8
8	Transport_Macropinocytosis regulation by growth factors	63	3.508E-05	12
9	Ovarian cancer (main signaling cascades)	64	4.134E-05	12
10	Expression targets of Tissue factor signaling in cancer	22	4.929E-05	7

E

Enrichment analysis report				
Enrichment by Pathway Maps		CX-5461, 24 hours		
#	Maps	Total	p-value	In Data
1	Immune response_IFN alpha/beta signaling pathway	24	1.681E-08	13
2	Development_Angiotensin signaling via STATs	32	1.202E-06	13
3	Protein folding and maturation_Angiotensin system maturation	43	1.819E-06	15
4	Transcription_Role of AP-1 in regulation of cellular metabolism	38	1.892E-06	14
5	Immune response_C5a signaling	50	3.057E-06	16
6	Development_Oligodendrocyte differentiation from adult stem c	51	4.096E-06	16
7	Cell adhesion_ECM remodeling	52	5.441E-06	16
8	Immune response_Antiviral actions of Interferons	52	5.441E-06	16
9	Immune response_IL-3 activation and signaling pathway	31	5.688E-06	12
10	Protein folding and maturation_Angiotensin system maturation	48	8.638E-06	15

Supplementary Figure S6: RNA-Sequencing differential gene expression analysis following inhibition of Pol I transcription initiation by CX-5461 and Act D. (**A**) BJ-T p53sh cells were treated with vehicle, 1 µM CX-5461 or 5 nM Act D for the indicated times. RNA was extracted and 47S rRNA precursor levels were determined. Expression levels were normalized to Vimentin mRNA and expressed as fold change relative to vehicle $t = 0$ ($n = 3$), error bars represent mean ± s.e.m, **p*-value < 0.05, ***p*-value < 0.01, compared to vehicle $t = 0$ samples. (**B**) BJ-T p53sh cells were treated with either vehicle (0.5 h), 1 µM CX-5461 for (0.5, 1, 3, 6, 12 and 24 h) or 5 nM Act D for (0.5 and 3 h). RNA was extracted from three biological replicates. Graphic representation of all differentially expressed genes with a false discovery rate less than 0.05 and $log_2FC \leq -1$ or $log_2FC \geq 1$. MetaCore gene ontology analysis of differentially expressed gene following 6-hour (**C**) 12-hour (**D**) and 24-hour (**E**) with 1 µM CX-5461. The most significantly enriched gene ontologies are represented. *p*-value denotes the significance of the number of differentially expressed genes (In Data) compared to the total number of genes (Total) in the gene ontology classification.

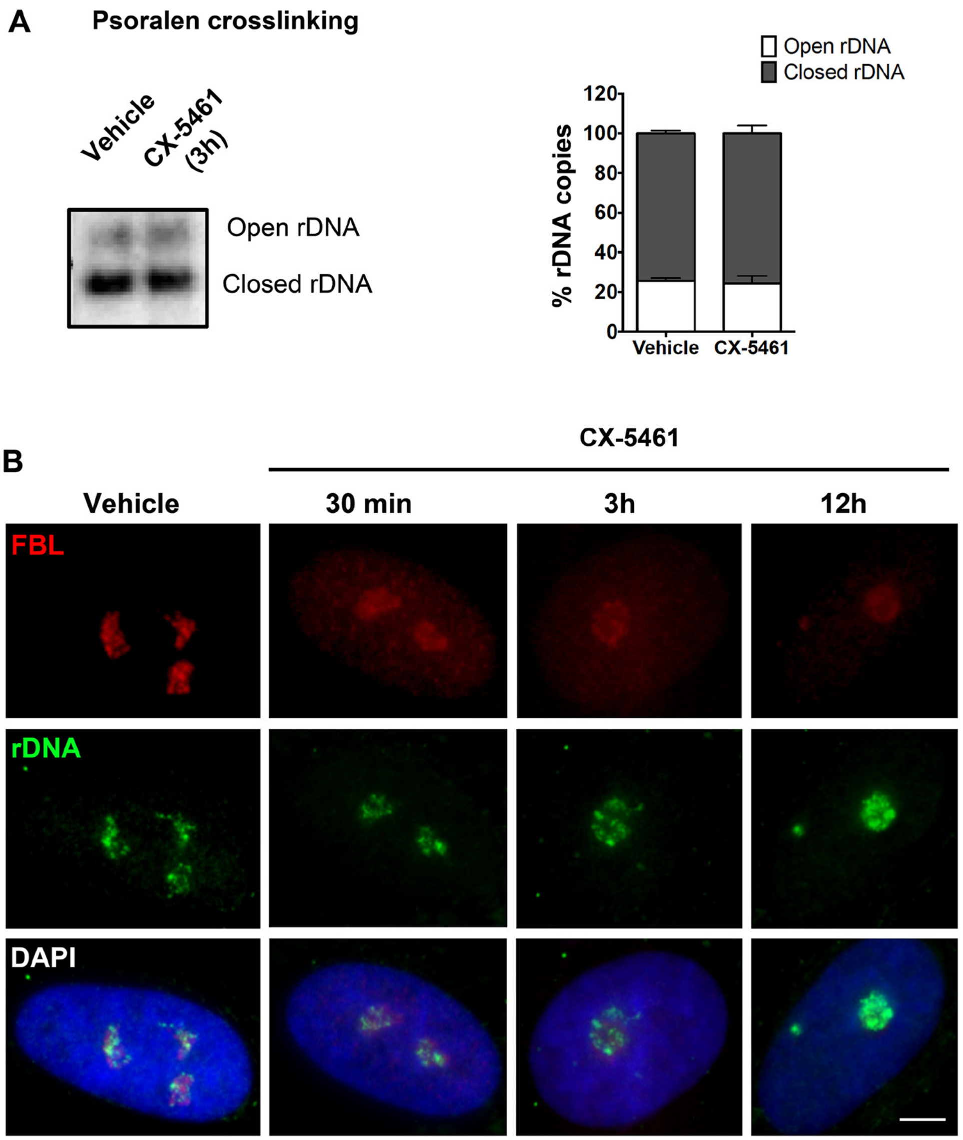

Supplementary Figure S7: (**A**) Inhibition of Pol I transcription initiation by CX-5461 results in rDNA repeats that are maintained in an exposed chromatin conformation. Nuclei from BJ-T cells treated with vehicle or 1 μM CX-5461 (3 h) were extracted and irradiated in the presence of psoralen. Genomic DNA was isolated and analyzed by Southern blotting for rDNA. The proportion of open versus closed rDNA was quantitated (right panel) using Phosphoimager Imagequant software ($n = 3$), error bars represent mean ± s.e.m. (**B**) CX-5461 treatment of BJ-T cells leads to the collapse of the nucleolar organiser regions (NORs) within the nucleoli. IF combined with FISH analysis for Fibrillarin (FBL) and rDNA with DAPI counterstain on BJ-T cells treated with 1 μM CX-5461 as indicated. Scale bar = 5 μM.

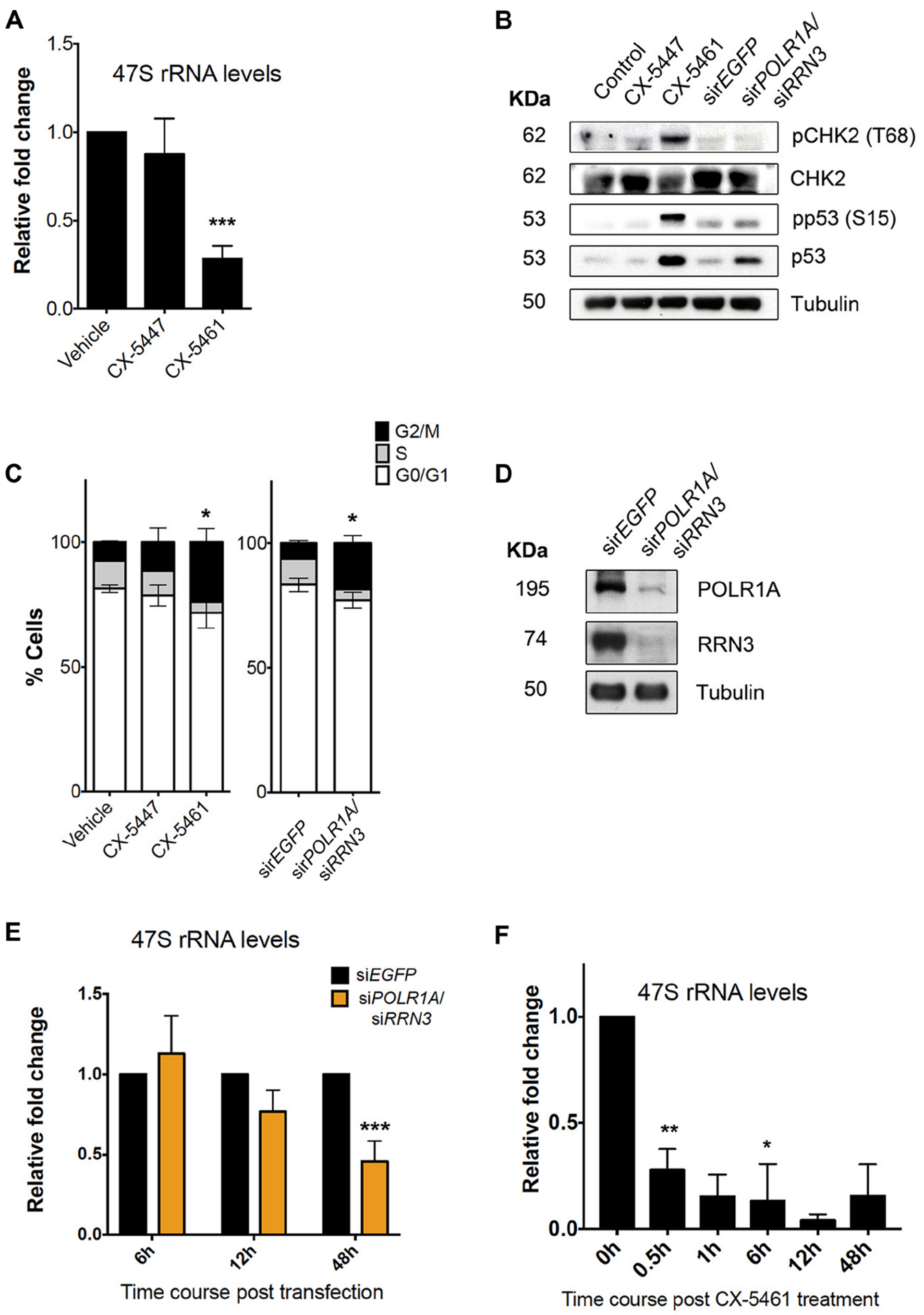

Supplementary Figure S8: (**A**) BJ-T cells were treated with for 48 h with 1 μM CX-5447 or CX-5461. Total RNA was extracted and 47S rRNA precursor levels were determined by reverse transcription qPCR. RNA levels were normalized to Vimentin mRNA and expressed as fold change relative to the vehicle treated controls, ($n = 4$) error bars represent mean ± s.e.m, ***$p < 0.001$ relative to control. (**B**) Western blot analysis of BJ-T cells were treated for 48 h with 1 μM CX-5447 or CX-5461 or transfected with sir*EGFP* and sir*POLR1A/RRN3* for 48 h, ($n = 3$). (**C**) Inhibition of Pol I transcription by CX-5461 ($n = 3$) and siPOLR1A/RRN3 knockdown ($n = 4$) led to a significant accumulation of cells in G2. Cell cycle analysis using PI staining of BJ-T cells treated as in (B). Error bars represent mean ± s.e.m, *$p < 0.05$ relative to corresponding controls. (**D**) Western blot analysis of BJ-T cells transfected with sir*EGFP* and sir*POLR1A/RRN3* for 48 h. Total protein lysates were extracted and western blot analysis was carried out with indicated antibodies against POLR1A, RRN3 and Tubulin. (**E**) BJ-T cells transfected with sir*EGFP* and sir*POLR1A/RRN3* were harvested and total RNA was extracted at 6 h, 12 h and 48 h post siRNA transfection. 47S rRNA precursor levels were determined by reverse transcription qPCR. RNA levels were normalized to Vimentin mRNA and expressed as fold change relative to the control samples (6 h and 12 h $n = 2$; 48 h $n = 6$), error bars represent mean ± s.e.m, ***p-value < 0.001 compared to corresponding control. (**F**) BJ-T cells were treated with 1 μM CX-5461 as indicated. Total RNA was extracted and 47S rRNA precursor levels were determined by reverse transcription qPCR. RNA levels were normalized to Vimentin mRNA and expressed as fold change relative to the control samples ($n = 2/3$), error bars represent mean ± s.e.m, *$p < 0.05$, **$p < 0.01$ compared to corresponding control.

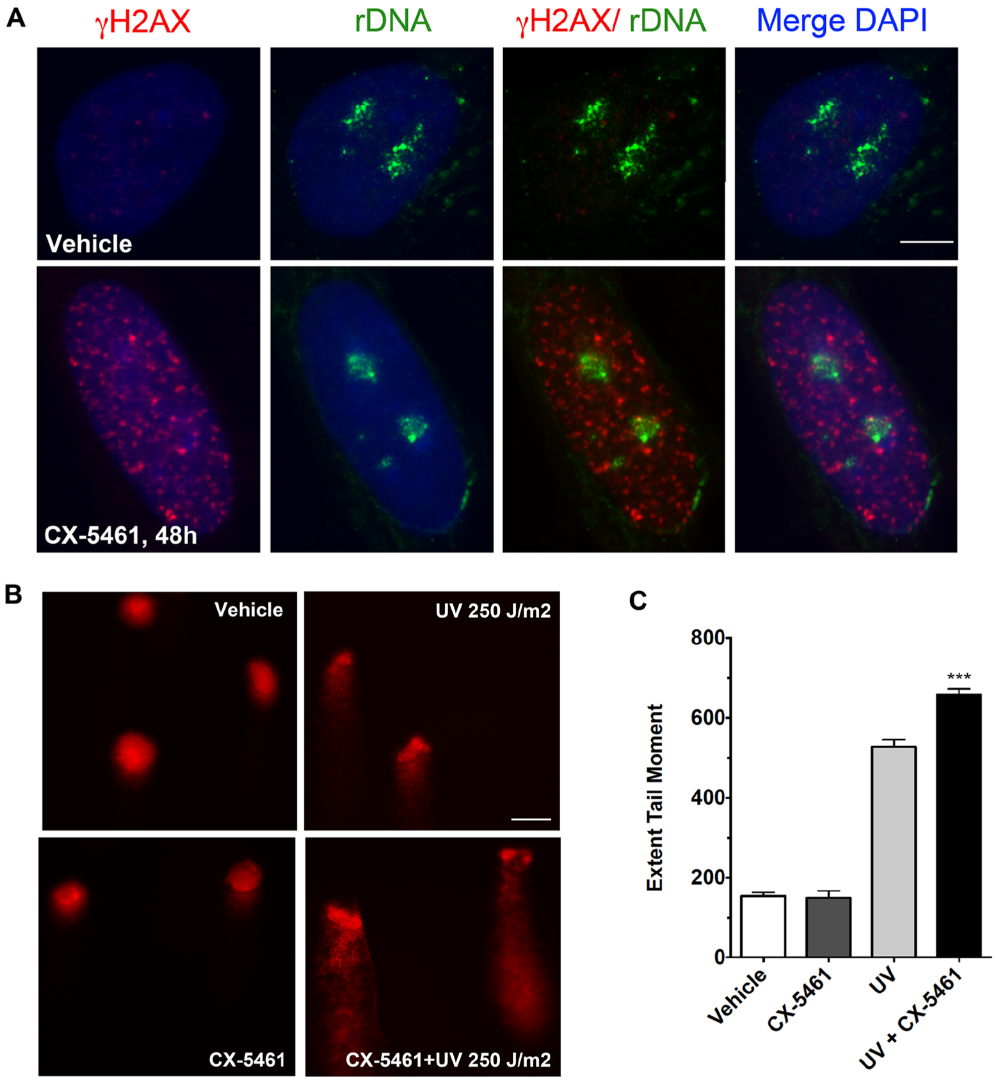

Supplementary Figure S9: (**A**) Chronic CX-5461 treatment leads to DNA damage outside rDNA loci. Combined IF-FISH analysis of γH2AX and rDNA in BJ-T cells treated with vehicle or 1 µM CX-5461 for 48 h. Scale bar = 5 µM. (**B**) CX-5461-mediated nucleolar stress attenuates DNA damage response. Alkaline comet assay analysis of DNA damage. BJ-T cells treated with either vehicle or 1 µM CX-5461 for 10 min in duplicates. One set of each treatment was exposed to 250 j/m2 UV irradiation and incubated for further 20 min. (Representative of n = 2). Scale bar = 20 µM. Quantitation of comet tails using Metamorph software is shown (right panel) for extent tail moment normalized to vehicle control. Error bars represent mean ± s.d.
